# Zinc finger protein 277 is an intestinal transit-amplifying cell marker and colon cancer oncogene

**DOI:** 10.1172/jci.insight.150894

**Published:** 2022-02-22

**Authors:** Guofeng Xie, Zhongsheng Peng, Jinqing Liang, Shannon M. Larabee, Cinthia B. Drachenberg, Harris Yfantis, Jean-Pierre Raufman

**Affiliations:** 1 VA Maryland Healthcare System, Baltimore, Maryland, USA.; 2 Department of Medicine, Division of Gastroenterology & Hepatology,; 3 Marlene and Stewart Greenebaum Comprehensive Cancer Center,; 4 Department of Surgery,; 5 Department of Pathology, and; 6 Department of Biochemistry and Molecular Biology, University of Maryland School of Medicine, Baltimore, Maryland, USA.

**Keywords:** Gastroenterology, Oncology, Cellular senescence, Colorectal cancer

## Abstract

Sustained proliferative signaling and resisting cell death are hallmarks of cancer. Zinc finger protein 277 (*ZNF277*; murine *Zfp277*), a transcription factor regulating cellular senescence, is overexpressed in colon cancer, but its actions in intestinal homeostasis and neoplasia are unclear. Using human and murine intestine, human colon cancer cells, and *Apc^Min/+^* mice with dysregulated **β**-catenin signaling and exuberant intestinal neoplasia, we explored the actions of *ZNF*2*77/Zfp277* and defined the underlying mechanisms. In normal human and murine intestine, *ZNF*2*77/Zfp277* was expressed uniquely in early stem cell progenitors, undifferentiated transit-amplifying cells (TACs). *Zfp277* was overexpressed in the *Apc^Min/+^* mouse colon, implicating *ZNF*2*77/Zfp277* as a transcriptional target of **β**-catenin signaling. We confirmed this by showing **β**-catenin knockdown reduced *ZNF277* expression and, using chromatin IP, identified 2 **β**-catenin binding sites in the *ZNF277* promoter. *Zfp277* deficiency attenuated intestinal epithelial cell proliferation and tumor formation, and it strikingly prolonged *Apc^Min/+^* mouse survival. RNA-Seq and PCR analyses revealed that *Zfp277* modulates expression of genes in key cancer pathways, including **β**-catenin signaling, the *HOXD* family that regulates development, and p21^WAF1^, a cell cycle inhibitor and tumor suppressor. In both human colon cancer cells and the murine colon, *ZNF*2*77/Zfp277* deficiency induced p21^WAF1^ expression and promoted senescence. Our findings identify *ZNF*2*77/Zfp277* as both a TAC marker and colon cancer oncogene that regulates cellular proliferation and senescence, in part by repressing p21^WAF1^ expression.

## Graphical abstract

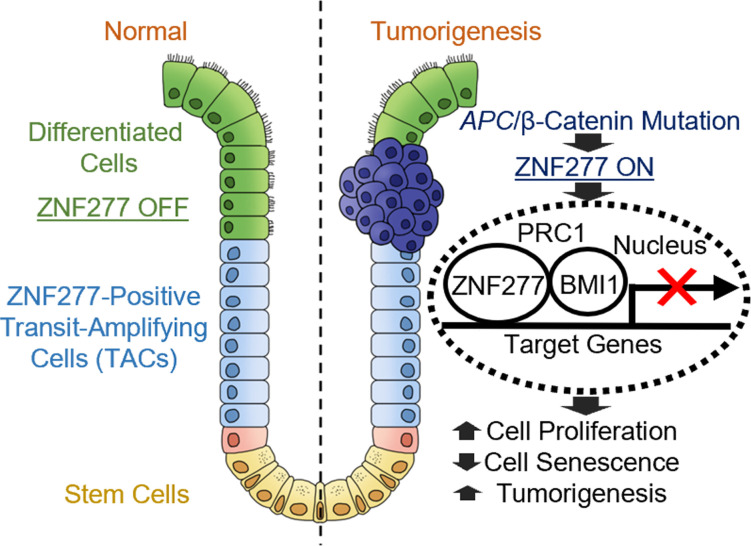

## Introduction

Despite advances in screening and treatment, in the United States, colorectal cancer (CRC) is responsible for approximately 50,000 deaths yearly and remains the second leading cause of cancer death (1). Chemoprevention with nonsteroidal antiinflammatory drugs including sulindac and aspirin has limited efficacy (2, 3) and may result in gastrointestinal (4) and cardiovascular toxicity (5, 6). Surgery, chemotherapy, and radiation for advanced metastatic disease also have limited efficacy. Although biologicals targeting vascular endothelial growth factor and the epidermal growth factor receptor (EGFR) may improve outcomes, they only modestly improve 5-year survival (~10%) (7–9). Newer immunotherapies benefit only a small subset of people with mismatch-repair-deficient colon cancer (10, 11). In addition to limited efficacy, use of these agents is limited by off-target toxicity that reduces their tolerance; for example, treatment targeted at EGFR, which is expressed widely in nonintestinal epithelial cells — e.g., dermal epithelial cells (12) — may cause severe skin reactions. These therapeutic limitations highlight the urgent need to identify novel molecular targets and approaches.

In the course of exploring the role of muscarinic cholinergic receptor expression and activation in colorectal neoplasia, we learned that the subtype 3 muscarinic receptor (M_3_R) is overexpressed in colon cancer, and — in both cell and animal models — reducing M_3_R expression or activation attenuates cancer cell proliferation, migration and invasion, and intestinal tumor formation (13–16). Using a microarray approach to compare changes in gene expression in colon tumors from WT and M_3_R-deficient mice, we found a zinc finger protein, *Zfp277*, was selectively downregulated in colon tumors from M_3_R-deficient mice with reduced tumorigenesis (17). By binding to DNA, RNA, protein, or other small molecules, zinc finger protein transcription factors play key roles in regulating gene expression and thereby contribute to a variety of biological processes, including cell proliferation, differentiation, apoptosis, and metabolism (18). The human homologue of *Zfp277*, *ZNF277*, is a classic C2H2 zinc finger gene highly conserved in humans, mice, zebra fish, *Drosophila*, and *C*. *elegans* (19). Human ZNF277 and murine Zfp277 share 5 zinc finger domains and a 30–amino acid coiled-coil domain. Murine Zfp277 is expressed in early embryonic stem cells, suggesting it may be a critical regulator of cell proliferation, differentiation, and cell fate transitions (20). Herein, we refer to *ZNF*2*77/Zfp277* when addressing attributes relevant to both the human and murine genes and proteins.

*ZNF*2*77/Zfp277* may play important roles in senescence; Negishi et al. showed that loss of Zfp277 from mouse embryonic fibroblasts prematurely induced senescence (21). Cellular senescence is characterized by proliferative arrest and the secretion of proteins that comprise the senescence-associated secretory phenotype (22). Besides impacting aging, senescence plays an important role in cancer. Although senescent cells are initially protected against neoplastic transformation, at later stages, they may contribute to a protumorigenic microenvironment (23, 24). Although no single biomarker can identify senescent cells, cyclin-dependent kinase inhibitor 1A (CDKN1A; p21^WAF1^), β-galactosidase, CDKN2A (also known as p16^INK4A^ and p19^ARF^ [mouse]/p14^ARF^[human]), and CDKN2B (INK4B; p15^INK4B^) are commonly associated with senescence (22).

In the current study, we explored the role of *ZNF*2*77/Zfp277* in normal intestinal epithelial cell development, proliferation, and senescence. Additionally, we used human colon cancer cells, and animal and enteroid models, to elucidate the mechanisms whereby *ZNF*2*77/Zfp277* overexpression promotes colon neoplasia. In doing so, we identified ZNF277 as a transcriptional target of β-catenin signaling that modulates Wnt/β-catenin and polycomb protein signaling, and it represses p21^WAF1^ expression, thereby regulating intestinal epithelial cell proliferation, senescence, and neoplasia. Overall, our findings uncover *ZNF*2*77/Zfp277* as a potentially novel intestinal transit-amplifying cell (TAC) marker and colon cancer oncogene.

## Results

### In normal intestinal epithelium, ZNF277/Zfp277 is only expressed in TACs.

**Figure 1:**
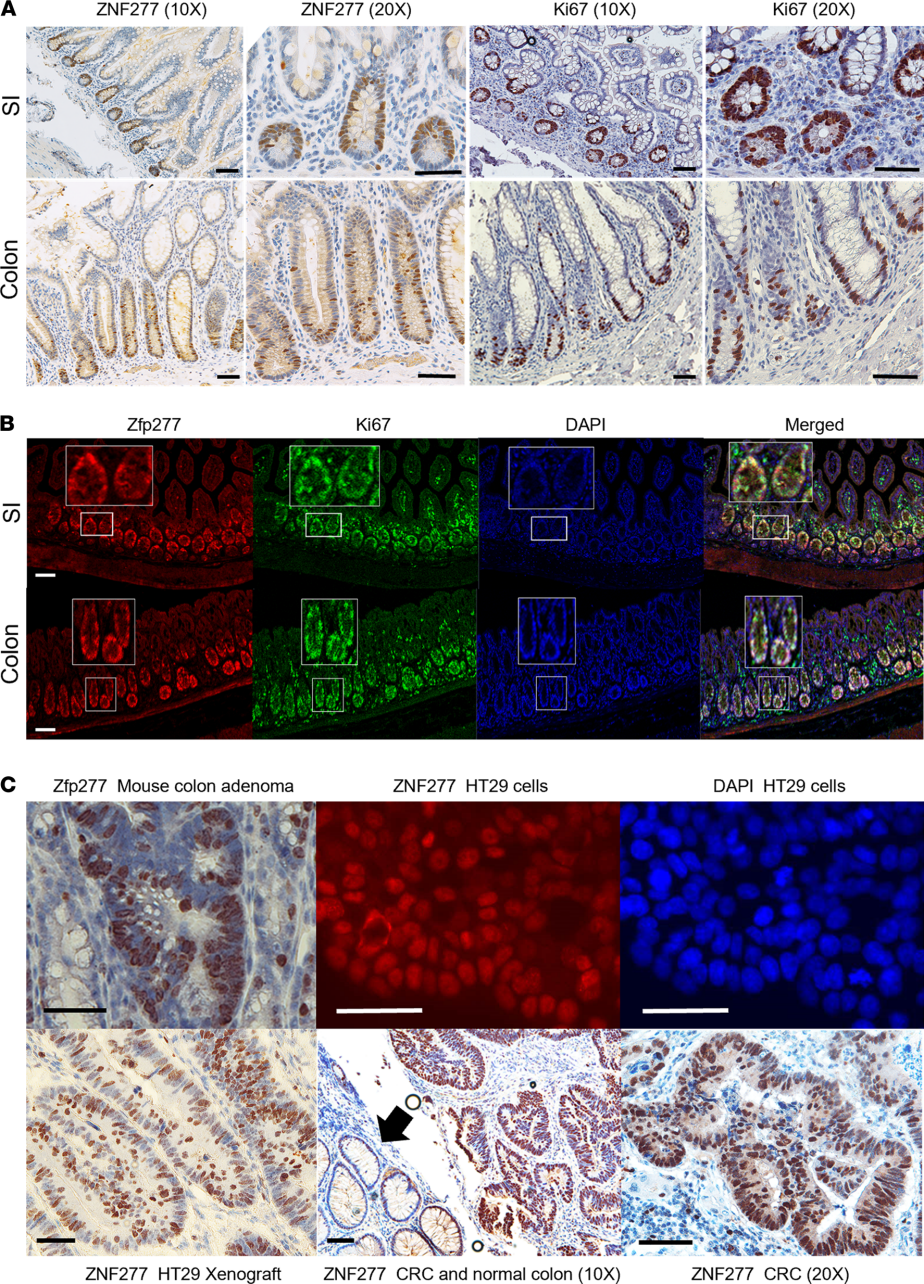
Expression and localization of ZNF277/Zfp277 in normal and neoplastic human and murine small intestine and colon. (**A**) In human small intestine (SI) and colonic crypts, ZNF277 is expressed selectively in the nuclei of transit amplifying cells (TACs). In the human ileum, IHC staining reveals ZNF277 and Ki67 expression in TACs. IHC reveals ZNF277 and Ki67 expression in transverse colon TACs. (**B**) Murine small intestinal and colonic TACs coexpress Zfp277 and Ki67. Immunofluorescence (IF) staining reveals Zfp277 and Ki67 expression in mouse ileal TACs and DAPI nuclear stains of mouse ileum. Merged Zfp277, Ki67, and DAPI confocal images reveal colocalization of Zfp277 and Ki67. IF staining reveals Zfp277 and Ki67 expression in murine colonic TACs. Merged Zfp277, Ki67, and DAPI images reveal Zfp277 and Ki67colocalization. (**C**) Nuclear localization of ZNF277/Zfp277 by IHC and IF staining. IHC reveals nuclear Zfp277 expression in a colon adenoma from an *Apc^Min/+^* mouse. IF staining reveals nuclear ZNF277 expression in HT29 human colon cancer cells. DAPI staining of HT29 cell nuclei. IHC reveals nuclear ZNF277 expression of cells in an HT29 cell xenograft, nuclear ZNF277 expression in human colon cancer and adjacent normal colon (arrow). Higher-magnification image showing nuclear ZNF277 expression in human colon cancer. Size bars: 100 μM.

In normal human small intestinal and colonic mucosa, we detected ZNF277 protein expression only in early stem cell progenitors — i.e., undifferentiated proliferating TACs (Figure 1A). Rapidly cycling TACs, identified by immunostaining for the cell proliferation marker Ki67, are localized to the lower half of intestinal crypts (Figure 1A). As shown in Figure 1B, in murine small intestine and colon, Zfp277 and Ki67 colocalize in TACs. We used murine small intestinal organoids (enteroids) to confirm and extend these findings. In murine enteroids, both Zfp277 and Ki67 were expressed selectively in nuclei of basal compartment cells where stem and TA cells reside. In contrast, β-catenin was prominently expressed in the cytoplasm and membranes of all enteroid cells (Supplemental Figure 1; supplemental material available online with this article; https://doi.org/10.1172/jci.insight.150894DS1). These results indicate that Zfp277 is selectively expressed in murine intestinal TACs but not in differentiated enterocytes.

### Murine TACs express 2 Zfp277 transcript variants.

Multiple transcript isoforms with variable functions can exist in mammalian genes. In mice, 2 *Zfp277* variants result from alternative splicing. Compared with Variant 1 (2454 bp; protein isoform 1; 583 aa), Variant 2 (2076 bp; protein isoform 2; 457 aa) lacks an alternate in-frame exon. Both variants share the same first 125 bp sequence (exon 1). Isoform 1 has an extra 378 bp from bp 126 to 504. We designed PCR primer pairs with the forward sequence within the first shared 125 bp sequence and the reverse primers between bp 504 and 602 (primer sequences in Methods). Variant 1 and 2 reverse transcription (RT)-PCR products resulting from the use of cDNAs prepared from WT mouse colon and enteroids were 595 and 309 bp in length, respectively (Supplemental Figure 2, A–C), and we detected both variants in colon mucosa and enteroids prepared from WT mice. In *Zfp277^–/–^* mice, Variant 1 is shorter due to removal of exon 5 in the *Zfp277*-KO transgenic mouse strain, and its expression is reduced compared with WT animals (Supplemental Figure 2A). Compared with WT mice, Variant 2 levels were also reduced (Supplemental Figure 2B). We detected the longer 65 kDa Zfp277 isoform 1 in murine liver (Supplemental Figure 3A) and the shorter isoform 2 (54 kDa) in mouse colon. In addition to the primary transcript (451 aa) detected in human colon cancer cells, there are 2 shorter human ZNF277 isoforms; it is unclear whether these 2 transcripts have different intestinal functions and are expressed at different stages of development — future studies will explore these possibilities. Next, to pursue our observation that *Zfp277* was selectively downregulated in colon tumors from M_3_R-deficient mice with attenuated tumorigenesis (17), we examined the role of ZNF277/Zfp277 in colon neoplasia.

### ZNF277/Zfp277 overexpression in neoplastic colon cell nuclei.

As shown in Supplemental Figure 4, in silico analysis using publicly available online databases — Oncomine (25), the Gene Expression Profiling Interactive Analysis (GEPIA) (26), and the Human Protein Atlas (27) — revealed increased ZNF277 mRNA and protein levels in colon cancer compared with normal colon. As shown in Supplemental Figure 5, in silico analysis using the UALCAN server (28) revealed that, regardless of sex, race, age, tumor stage, and nodal metastasis, *ZNF277* transcript levels are 1.5- to 2.0-fold greater in tumors compared with normal tissues. Using data from the GEPIA server, we detected modest but statistically significant correlations between *ZNF277* expression levels and *BRAF*, *APC*, and p21 (*CDKN1A*) genetic subtypes (*R* = 0.39 and *P* = 8.9 × 10^–13^, *R* = –0.19 and *P* = 0.00067, and *R* = –0.26 and *P* = 2.5 × 10^–6^, respectively); there was no correlation between *ZNF277* and *p53* levels (*R* = 0.016, *P* = 0.77). Analyzing data extracted from the Colon Adenocarcinoma (COAD) data set (GEPIA), we did not detect a relationship between *ZNF277* levels and CRC survival. Because ZNF277 is expressed in many tissues, including the immune system, ZNF277 expression levels in tumor cells alone might not be sufficient to alter survival. IHC staining of murine adenomas, human colon cancer cells, colon cancer cell xenografts, and colon cancers revealed that ZNF277/Zfp277 protein overexpression was localized to cell nuclei (Figure 1C).

### Zfp277 deficiency attenuates adenoma formation and progression in Apc^Min/+^ mice.

To elucidate the role of Zfp277 expression in intestinal tumorigenesis and progression, we obtained *Zfp277^+/–^* (B6.129-Zfp277<tm1>) mice wherein deletion of exon 5 of the *Zfp277* gene results in complete loss of Zfp277 protein expression (21). Homozygous mutant mice backcrossed to the C57BL/6J genetic background were healthy and fertile (21). Zfp277 protein deficiency was confirmed by immunoblotting (see Methods).

**Figure 2:**
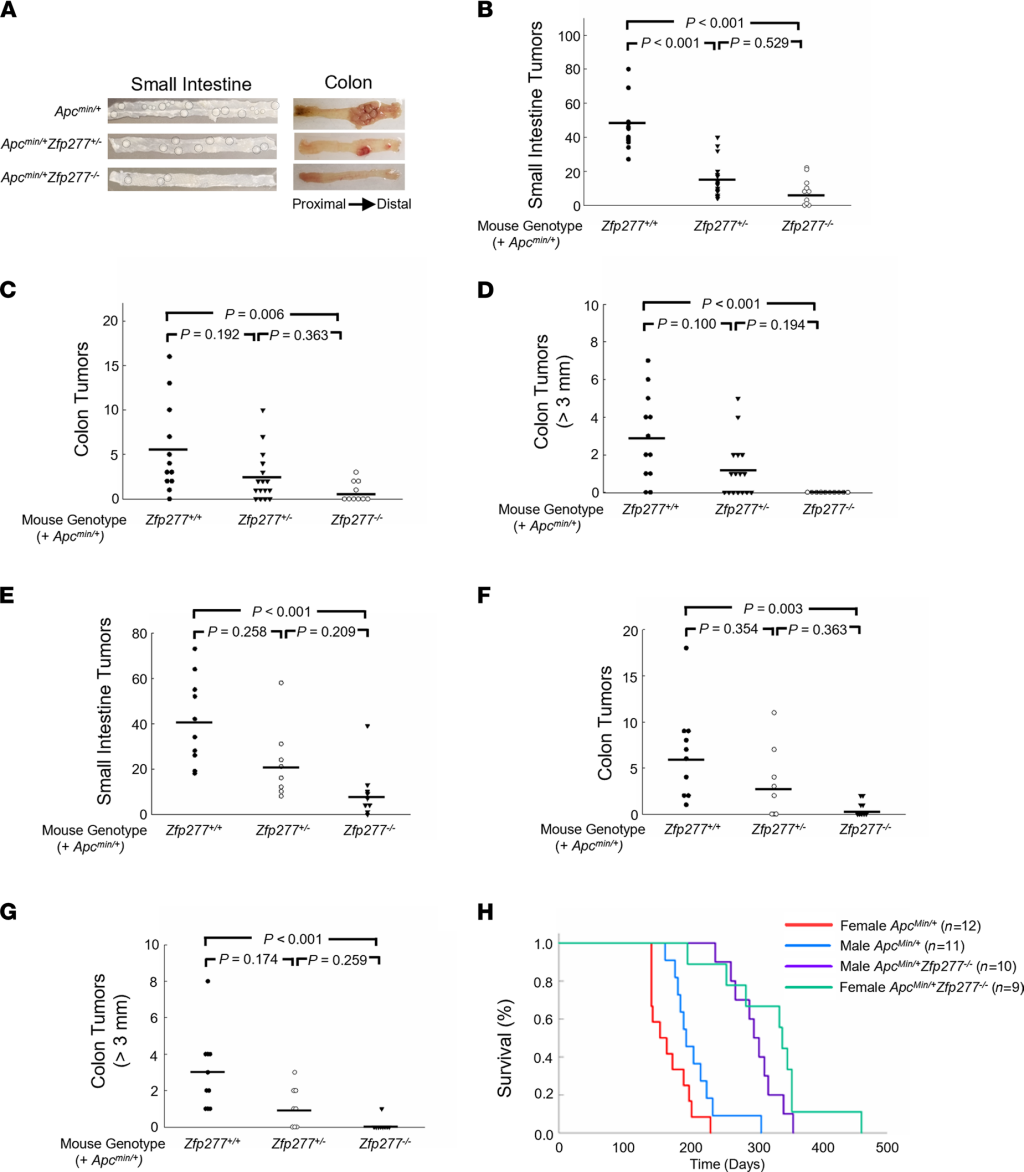
*Zfp277* deficiency attenuates intestinal neoplasia and prolongs the survival of *Apc^Min/+^* mice. (**A**) Representative images of the proximal small intestine and colon of 15-week-old male mice with the indicated genotypes. (**B**–**D**) *Zfp277* deficiency reduces the number of small intestine (**B**) and colon (**C** and **D**) tumors in male *Apc^Min/+^* mice. Twelve *Apc^Min/+^ Zfp277^+/+^*, 16 *Apc^Min/+^ Zfp277^+/–^,* and 10 *Apc^Min/+^ Zfp277^–/–^* mice were used. (**E**–**G**) *Zfp277* deficiency reduces the number of small intestine (**E**) and colon (**F** and **G**) tumors in female *Apc^Min/+^* mice. Ten *Apc^Min/+^ Zfp277^+/+^*, 8 *Apc^Min/+^ Zfp277^+/–^*, and 9 *Apc^Min/+^ Zfp277^–/–^* mice were used. Each symbol represents 1 mouse. Horizontal bars represent mean values. Statistical analysis was performed using 1-way ANOVA with Dunn’s tests. (**H**) Kaplan-Meier survival analysis of male and female *Apc^Min/+^* and *Apc^Min/+^ Zfp277^–/–^* mice.

To examine the effects of *Zfp277* deficiency in *Apc^Min/+^* mice with an *Apc* gene mutation that results in dysregulated β-catenin signaling and the development of small and large intestinal adenomas, we created *Apc^Min/+^* mice with heterozygous and homozygous *Zfp277* deficiency. Because of sex differences in human colon neoplasia, we explored the effects of *Zfp277* deficiency in both male and female *Apc^MIn/+^* mice. As shown in Figure 2, A–G, the numbers of small intestine and colon tumors were substantially reduced in both male (Figure 2, A–D) and female (Figure 2, E–G) *Apc^Min/+^ Zfp277^+/–^* and *Apc^Min/+^ Zfp277^–/–^* mice compared with *Apc^Min/+^ Zfp277^+/+^* littermate control mice. Male *Apc^Min/+^ Zfp277^+/+^* (*n* = 12), *Apc^Min/+^ Zfp277^+/–^* (*n* = 16), and *Apc^Min/+^ Zfp277^–/–^* (*n* = 10) mice had 46.0 ± 4.3, 16.4 ± 2.7, and 8.6 ± 2.6 (mean ± SEM) small intestinal tumors, respectively (Figure 2B). The significantly reduced small intestinal tumor burden in male *Apc^Min/+^* mice with *Zfp277* haploinsufficiency supports an important role for this gene in intestinal neoplasia.

Most tumors were localized to the distal small intestine, and in each small intestinal segment, fewer tumors were observed in *Apc^Min/+^ Zfp277^–/–^* compared with *Apc^Min/+^ Zfp277^+/+^* mice (Supplemental Figure 6A). Although — as is typical for *Apc^Min/+^* mice — there were fewer colon tumors, *Zfp277* deficiency also reduced the number of colon tumors; male *Apc^Min/+^ Zfp277^+/+^*, *Apc^Min/+^ Zfp277^+/–^,* and *Apc^Min/+^ Zfp277^–/–^* mice had 5.5 ± 1.4, 2.4 ± 0.7, and 0.8 ± 0.3 (mean ± SEM) colon tumors, respectively (Figure 2C). As shown in Figure 2D, in *Apc^Min/+^ Zfp277^–/–^* mice, colon tumors were much smaller than those in *Apc^Min/+^* *Zfp277^+/+^* littermates; in contrast to *Apc^Min/+^ Zfp277^+/+^* and *Apc^Min/+^ Zfp277^+/–^* mice, no *Apc^Min/+^ Zfp277^–/–^* mice had colon tumors greater than 3 mm in largest diameter (Figure 2D).

Likewise, as shown in Figure 2, E–G, *Zfp277* deficiency attenuated intestinal tumor formation in female mice. Female *Apc^Min/+^* *Zfp277^+/+^* (*n* = 10), *Apc^Min/+^ Zfp277^+/–^* (*n* = 8), and *Apc^Min/+^* *Zfp277^–/–^* mice (*n* = 9) had 41.1 ± 6.1, 22.5 ± 5.7, and 9.6 ± 3.9 (mean ± SEM) small intestinal tumors, respectively (Figure 2E). Again, most tumors were in the distal small intestine and, in each small intestinal segment, fewer tumors were found in *Apc^Min/+^* *Zfp277^–/–^* compared with *Apc^Min/+^ Zfp277^+/+^* mice (Supplemental Figure 6B).

Zpf277 deficiency reduced the number of colon tumors 10-fold from 6.6 ± 1.6 tumors (mean ± SEM) in *Apc^Min/+^* *Zfp277^+/+^* mice to 3.4 ± 1.4 and 0.7 ± 0.3 tumors in *Apc^Min/+^ Zfp277^+/–^* and *Zfp277^–/–^ Apc^Min/+^* littermates, respectively (Figure 2F). Whereas *Apc^Min/+^* *Zfp277^+/+^* mice had 3.0 ± 0.7 colon tumors that were 3 mm in diameter or larger, *Apc^Min/+^ Zfp277^+/–^* and *Apc^Min/+^ Zfp277^–/–^* mice had only 1.1 ± 0.4 and 0.1 ± 0.1 tumors within this size range, respectively (Figure 2G). These striking changes in both tumor number and size support a role for Zfp277 in both tumor initiation and progression.

### Zfp277 deficiency substantially prolongs Apc^Min/+^ mouse survival.

As shown in Supplemental Figure 6C, by 15 weeks of age, whereas female *Apc^Min/+^* *Zfp277^–/–^* mice weighed the same as *Apc^+/+^* *Zfp277^+/+^* mice, they weighed significantly more than *Apc^Min/+^* *Zfp277^+/+^* mice. As shown in Supplemental Figure 6D, at 15 weeks, whereas *Apc^+/+^* *Zfp277^+/+^*, *Zfp277^–/–^*, and *Apc^Min/+^* *Zfp277^–/–^* mice had normal hematocrits, both male and female *Apc^Min/+^* littermate mice had become severely anemic.

As shown in Figure 2H, both male and female *Apc^Min/+^* *Zfp277^–/–^* mice lived much longer than *Apc^Min/+^ Zfp277^+/+^* littermates; mean survival for male *Apc^Min/+^* *Zfp277^+/+^* mice (*n* = 11) was 207 days versus 299 days for male *Apc^Min/+^* *Zfp277^–/–^* mice (*n* = 10; *P* < 0.001). Notably, this survival advantage was even more striking for female *Zfp277*-deficient mice; mean survival for female *Apc^Min/+^* *Zfp277^+/+^* littermate controls (*n* = 12) was 168 days versus 325 days for female *Apc^Min/+^* *Zfp277^–/–^* mice (*n* = 9) — an almost 2-fold difference (*P* < 0.001). In contrast, the mean survival of male *Apc^Min/+^* and female *Apc^Min/+^* mice (*P* = 0.0692) and the survival of female versus male *Apc^Min/+^* *Zfp277^–/–^* mice were not significantly different (*P* = 0.21). Likewise, we did not observe differences in the survival of male and female WT or *Zfp277*-KO mice (data not shown).

### Zfp277 promotes intestinal epithelial cell proliferation.

**Figure 3:**
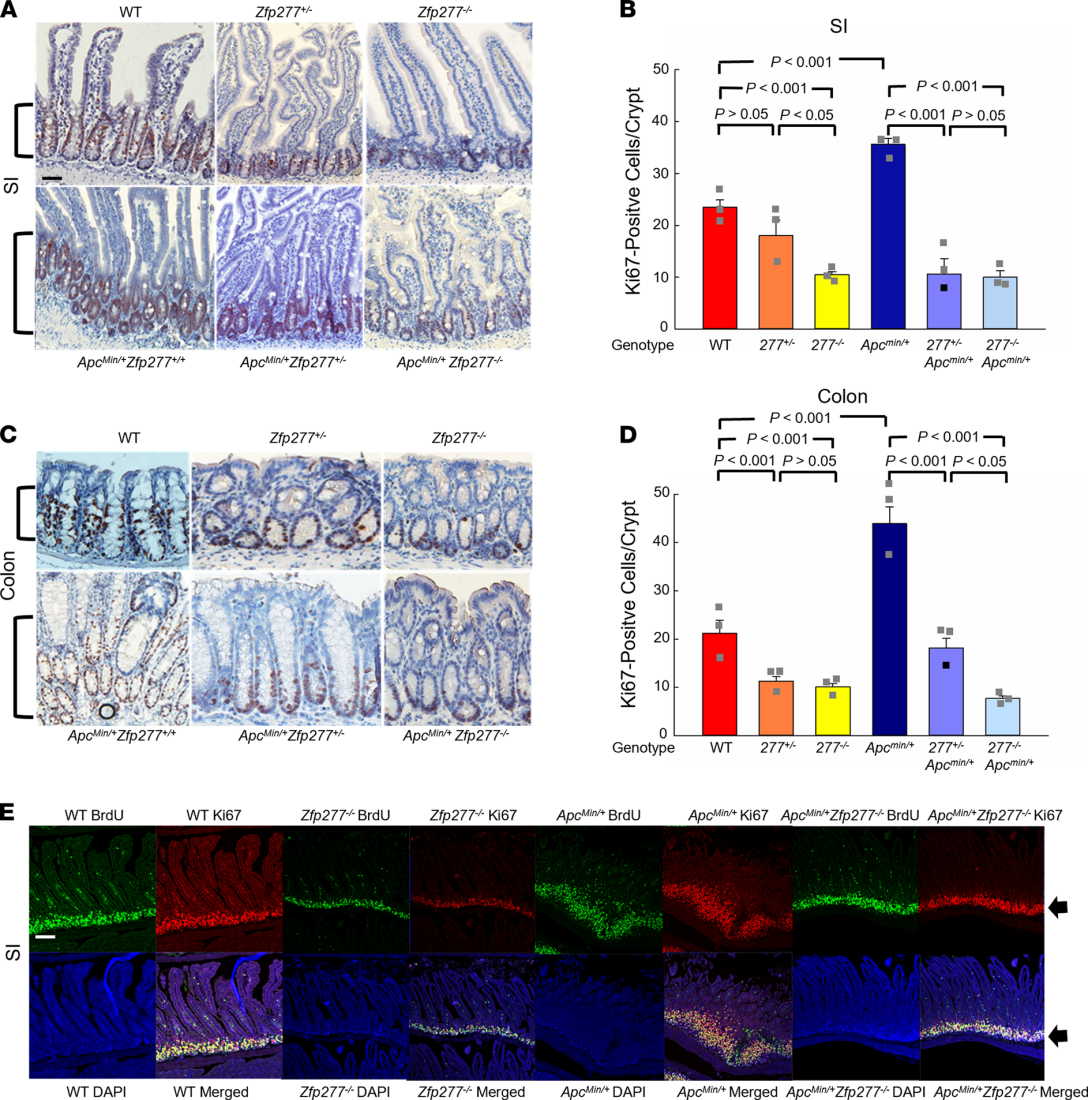
*Zfp277* deficiency attenuates epithelial cell proliferation in murine small intestine and colon. (**A**) *Zfp277* deficiency reduces small intestinal Ki67 expression. Brackets indicate staining at the base of the crypts. Representative Ki67 IHC staining of ileal segments from mice with the indicated genotypes. (**B**) Numbers of Ki67^+^ cells per ileal crypt in mice of the indicated genotype. (**C**) Representative Ki67 IHC staining of distal colons from mice with the indicated genotype. Values represent mean ± SEM from 3 mice for each genotype. (**D**) Numbers of Ki67^+^ cells per colon crypt. All mice were 15-week-old males. Scale bar: 100 μM. Values represent mean ± SEM from 3 mice in each genotype. Data were analyzed using 1-way ANOVA. (**E**) Representative confocal microscopy images of BrdU (green; 2-hour labeling) and Ki67 (red) IF staining and merged images of BrdU, Ki67, and DAPI of ileal segments from 15-week-old male mice with the indicated genotype. Arrows indicate fluorescence signals at the base of the crypts. Scale bar: 100 μM.

To determine if changes in cell proliferation or apoptosis played roles in attenuating tumorigenesis and progression in *Zfp277*-deficient mice, we measured (a) Ki67 and BrdU staining and (b) caspase-3 activation, respectively. We found markedly reduced indices of cell proliferation in both the small intestine (Figure 3, A and E) and colon (Figure 3C) of *Zfp277*-deficient mice. TAC proliferation, measured by BrdU staining, was reduced in both the small intestine (Figure 3B) and colon (Figure 3D) of *Zfp277*-deficient mice possessing either WT or mutated *Apc*. Zfp277 deficiency did not alter apoptosis; we detected similar numbers of apoptotic cells in adenomas and normal intestinal tissues from *Apc^Min/+^* and *Apc^Min/+^ Zfp277^–/–^* mice (Supplemental Figure 7).

### ZNF277 promotes cell proliferation in vitro and in vivo.

**Figure 4:**
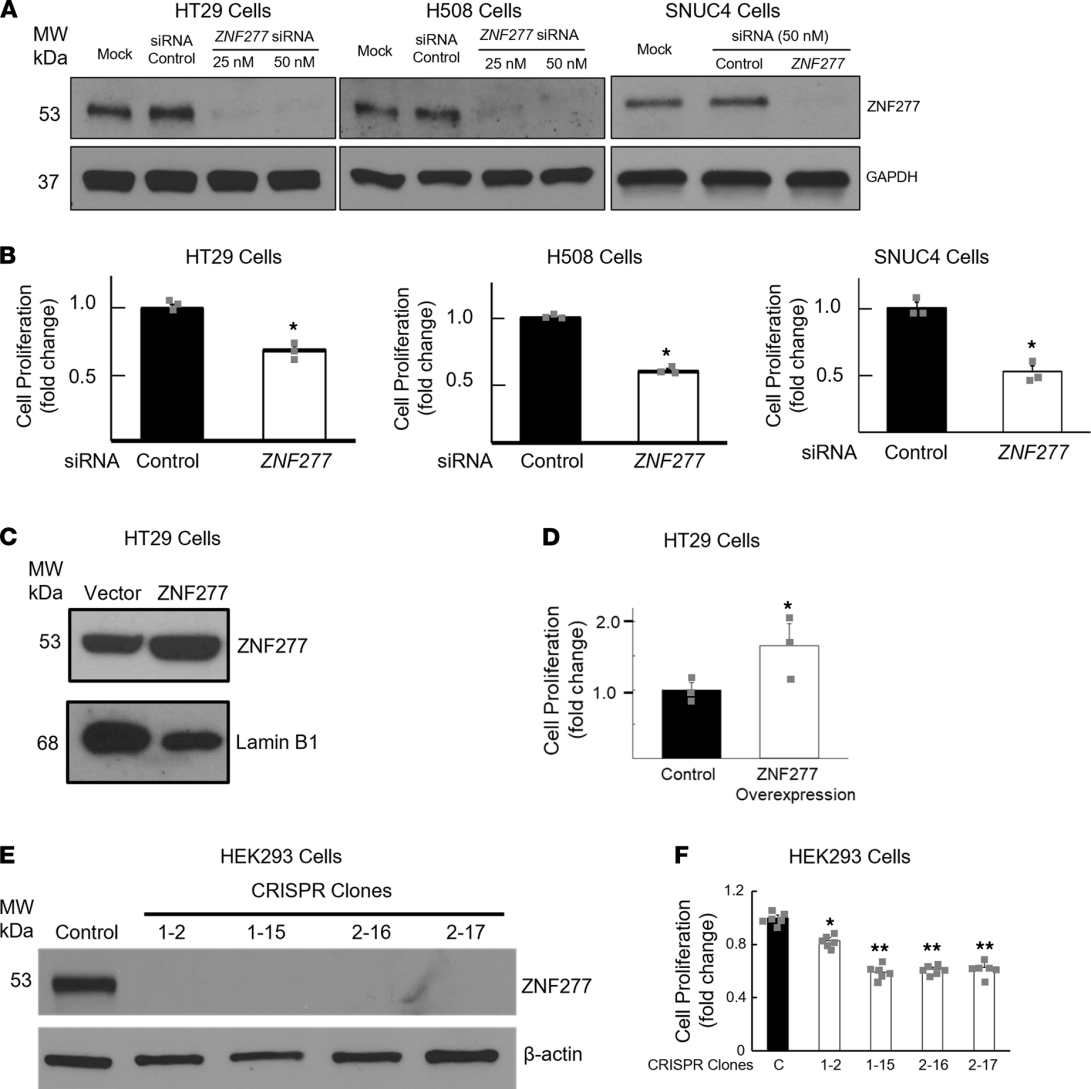
*ZNF277* promotes human colon cancer cell proliferation. (**A**) *ZNF277* RNA interference reduces ZNF277 protein expression in human colon cancer cells. Immunoblots of extracts from HT29, H508, and SNUC4 human colon cancer cells after *ZNF277* knockdown with the indicated concentrations of *ZNF277* siRNA and 50 nM mock siRNA. (**B**) *ZNF277* deficiency attenuates human colon cancer cell proliferation. Cells were transfected for 24 hours with siRNA, and cell proliferation was measured after an additional 24-hour incubation. *P < 0.05 versus control siRNA. Data represent mean ± SEM from 3 separate experiments. (**C**) Immunoblotting confirms ZNF277 overexpression in HT29 cells transfected with plasmid containing full-length human *ZNF277* cDNA. (**D**) Overexpressing *ZNF277* stimulates HT29 cell proliferation. *P < 0.05 versus control cells. Data represent mean ± SEM from 3 separate experiments. (**E**) Immunoblots reveal lack of ZNF277 expression in 4 HEK293 lines following CRISPR KO of *ZNF277*. (**F**) CRISPR KO of *ZNF277* attenuates HEK293 cell proliferation. *P < 0.05 versus control cells. **P < 0.05 versus line 1-2. Data are shown as mean ± SD from 7 separate experiments. Data were analyzed using 2-tailed *t* tests and 1-way ANOVA with post hoc Tukey test. β-Actin and lamin B1 were used as loading controls in **A** and **C**, respectively.

To gain additional functional and mechanistic insights into the role of *ZNF*2*77/Zfp277* , we examined the effects of modulating *ZNF277* expression in human colon cancer cells. As shown in Figure 4, A and B, in HT29, H508, and SNUC4 cells, small interfering RNA (siRNA) *ZNF277* knockdown inhibited in vitro cell proliferation. As anticipated from these findings, overexpressing *ZNF277* in HT29 cells increased cell proliferation (Figure 4, C and D). To determine whether *ZNF277* regulates cell proliferation in nonintestinal epithelial cells, we examined the effect of *ZNF277* knockdown on HEK293 cells, a commonly used kidney epithelial cell line. As shown in Figure 4, E and F, CRISPR KO of *ZNF277* using guide RNAs (gRNAs) significantly attenuated cell proliferation in HEK293 epithelial cells, indicating that *ZNF277* can regulate the proliferation of multiple epithelial cell types.

**Figure 5:**
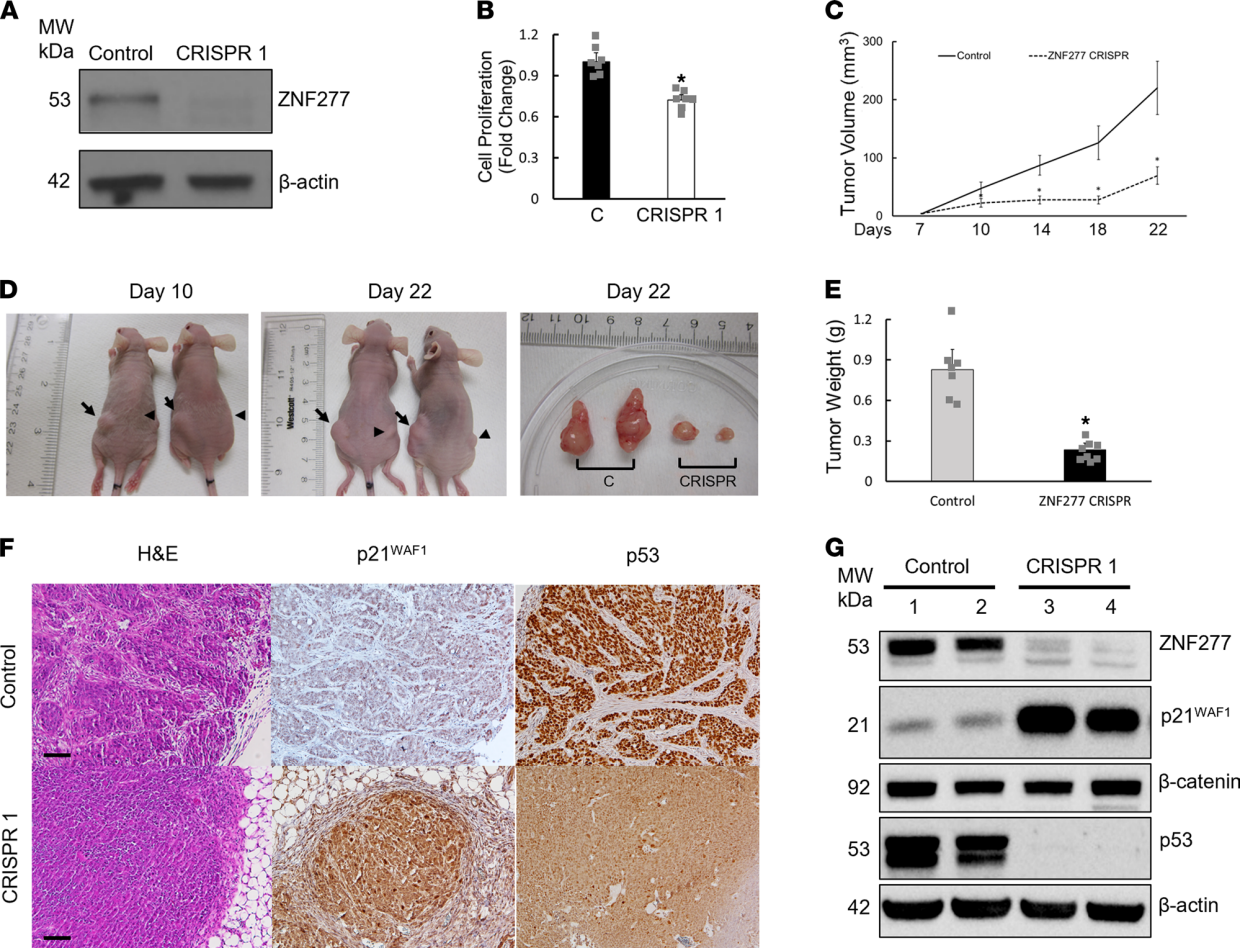
ZNF277 deficiency attenuates xenograft growth. (**A**) Immunoblots of HT29 cell extracts without (control) or with CRISPR knockdown of *ZNF277* expression. β-Actin was used as a loading control. (**B**) *ZNF277* deficiency attenuates HT29 cell proliferation in vitro from 7 separate experiments. (**C**) *ZNF277* deficiency attenuates xenograft growth. Time-course reveals reduced volume of *ZNF277* CRISPR HT29 cell–derived xenografts (*n* = 8) compared with control xenografts (*n* = 7). (**D**) Representative images of s.c. and excised xenografts from HT29 *ZNF277* CRISPR versus control cells. Arrows and arrowheads indicate control and HT29 *ZNF277* CRISPR xenografts, respectively. (**E**) Reduced weights of xenografts with *ZNF277* deficiency (*n* = 8). *P < 0.01 versus controls (*n* = 7); 2-tailed Student’s *t* test. (**F**) Representative microscopic images of control and *ZNF277*-deficient xenografts stained for H&E, p21^WAF1^, and p53. (**G**) ZNF277, p21^WAF1^, p53, and β-catenin immunoblots of proteins extracted from *ZNF277* CRISPR cell– and control cell–derived tumors (2 separate tumors from each group). Values represent mean ± SD. Scale bar: 100 μM.

To determine whether *ZNF277* deficiency affects cell proliferation in vivo, we examined the effect of CRISPR KO of *ZNF277* on the growth of human colon cancer cell xenografts. First, we generated a pooled *ZNF277*-KO cell line using *ZNF277* CRISPR gRNA constructs in HT29 cells (see Methods). As shown in Figure 5, A and B, respectively, CRISPR KO of *ZNF277* in pooled HT29 cells resulted in negligible ZNF277 protein expression and attenuated cell proliferation. Over the 3 weeks after cells were injected into the flanks of nude mice, compared with xenografts created using control cells, we observed greatly diminished *ZNF277* CRISPR xenograft volumes and weights (Figure 5, C–E). In addition — as shown in Figure 5, F and G, respectively — IHC and immunoblots revealed robustly increased p21^WAF1^ protein levels in xenografts generated from CRISPR *ZNF277*-KO cells. Interestingly, p53 protein expression was undetectable in xenografts with low *ZNF277* expression (Figure 5, F and G). Collectively, these results suggest that *ZNF277* promotes intestinal epithelial cell proliferation, in part by inhibiting p21^WAF1^ expression.

### ZNF277 is a transcriptional target of β-catenin.

**Figure 6:**
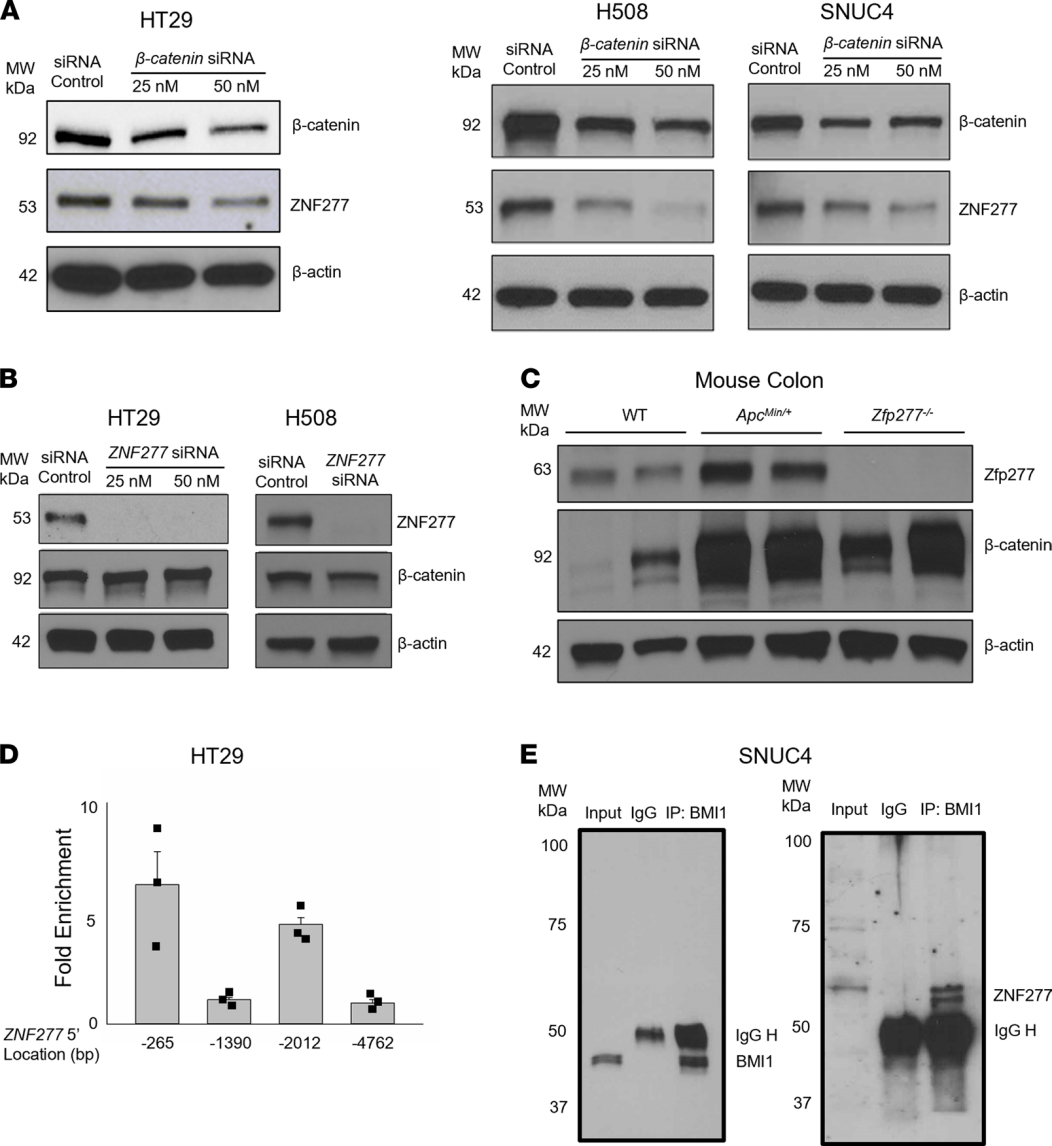
*ZNF277* expression is regulated by β-catenin and ZNF277 association with BMI1 in human colon cancer cells. (**A**) β-Catenin (*CTNNB1*) knockdown decreases ZNF277 levels in human HT29, H508, and SNUC4 colon cancer cells. (**B**) *ZNF277* knockdown does not alter β-catenin levels in HT29 and H508 colon cancer cells. Maximum siRNA concentration was 50 μM. (**C**) Increased β-catenin signaling augments Zfp277 expression. Zfp277 expression in colon mucosal extracts from WT, *Apc^Min/+^*, and *Zfp277^–/–^* mice. β-Actin was used as a loading control. (**D**) *ZNF277* promoter elements for β-catenin. ChIP assay using random DNA fragments generated by MNase digestion in HT29 cells. *ZNF277* promoter positions are indicated in the text and DNA sequences of qPCR primers are listed in Supplemental Table 2. Data are shown as mean ± SEM from 3 separate experiments. (**E**) ZNF277 coimmunoprecipitates with BMI1 in SNUC4 colon cancer cells. Rabbit immunoglobulins (IgG) were used as control.

The Wnt signaling pathway plays an instrumental role in regulating intestinal epithelial homeostasis; β-catenin is the most prominent downstream effector of Wnt signaling (29–32). To determine whether β-catenin regulates *ZNF277* expression, we examined the effect of β-catenin knockdown. As shown in Figure 6A, in 3 human colon cancer cell lines, siRNA knockdown of β-catenin (*CTNNB1*) dose-dependently reduced ZNF277 protein expression. In control experiments, *ZNF277* knockdown did not alter β-catenin expression in either HT29 or H508 cells (Figure 6B). As anticipated from these findings, Zfp277 protein levels were increased in the colons of *Apc^Min/+^* mice with activated β-catenin signaling (Figure 6C). Collectively, these results indicate that *ZNF277* expression is regulated by Wnt/β-catenin signaling.

Canonical actions of β-catenin require it to form a transcriptional complex with T cell factor (TCF) and lymphoid enhancing factor (LEF) (29). We used ChIP assays to determine if such a complex could regulate *ZNF277* transcription by binding to its promoter. Consensus TCF/LEF binding sites in human colon cancer cells contain the CTTTG(A/T) (A/T) sequence (33). Four consensus TCF/LEF binding sites are located within 5.0 kb of the 5′ *ZNF277* promoter from the transcriptional start site: 5′gctttgtaa at –265, 5′cctttgttg at –1390, 5′actttgaag at –2012 and 5′actttgtacactttgatc at –4762. To identify the binding site(s) for β-catenin/TCF/LEF in the *ZNF277* promoter in HT29 human colon cancer cells with robust β-catenin and *ZNF277* expression, we performed ChIP assays using quantitative PCR (qPCR) primers for each potential site (Supplemental Table 2) with ChIP-grade anti–β-catenin antibodies. As shown in Figure 6D, ChIP qPCR results indicated 6.5- and 4.5-fold enhanced binding by β-catenin at the –265 and –2012 sites, respectively, but we failed to detect enhanced binding at the other 2 sites. These results support the conclusion that, in human colon cancer cells, β-catenin regulates *ZNF277* transcription. Potential cotranscriptional factors of ZNF277 in TACs remain to be identified.

### ZNF277 is associated with B lymphoma Mo-MLV insertion region 1 homolog (BMI1).

In mouse embryonic fibroblasts, Negishi et al. showed physical association of Bmi1 with the N-terminal domain (1–58 aa) of Zfp277 (21). BMI1 is an important component of polycomb group complex 1 (PRC1). To determine whether ZNF277 interacts physically with BMI1 in humans, we performed co-IP using protein extracts from SNUC4 human colon cancer cells; these experiments showed robust ZNF277 and BMI1 protein expression. As shown in Figure 6E, we detected abundant ZNF277 in BMI1 immunoprecipitates, indicating that ZNF277 forms a protein complex with BMI1. This finding suggests that ZNF277 is a component of the PRC1 protein complex in human colon cancer cells. In addition, this physical ZNF277-BMI1 association provides a potential target for small molecule inhibitors to disrupt this protein-protein interaction.

### ZNF277 inhibits cellular senescence in vitro by repressing p21^WAF1^ expression.

**Figure 7:**
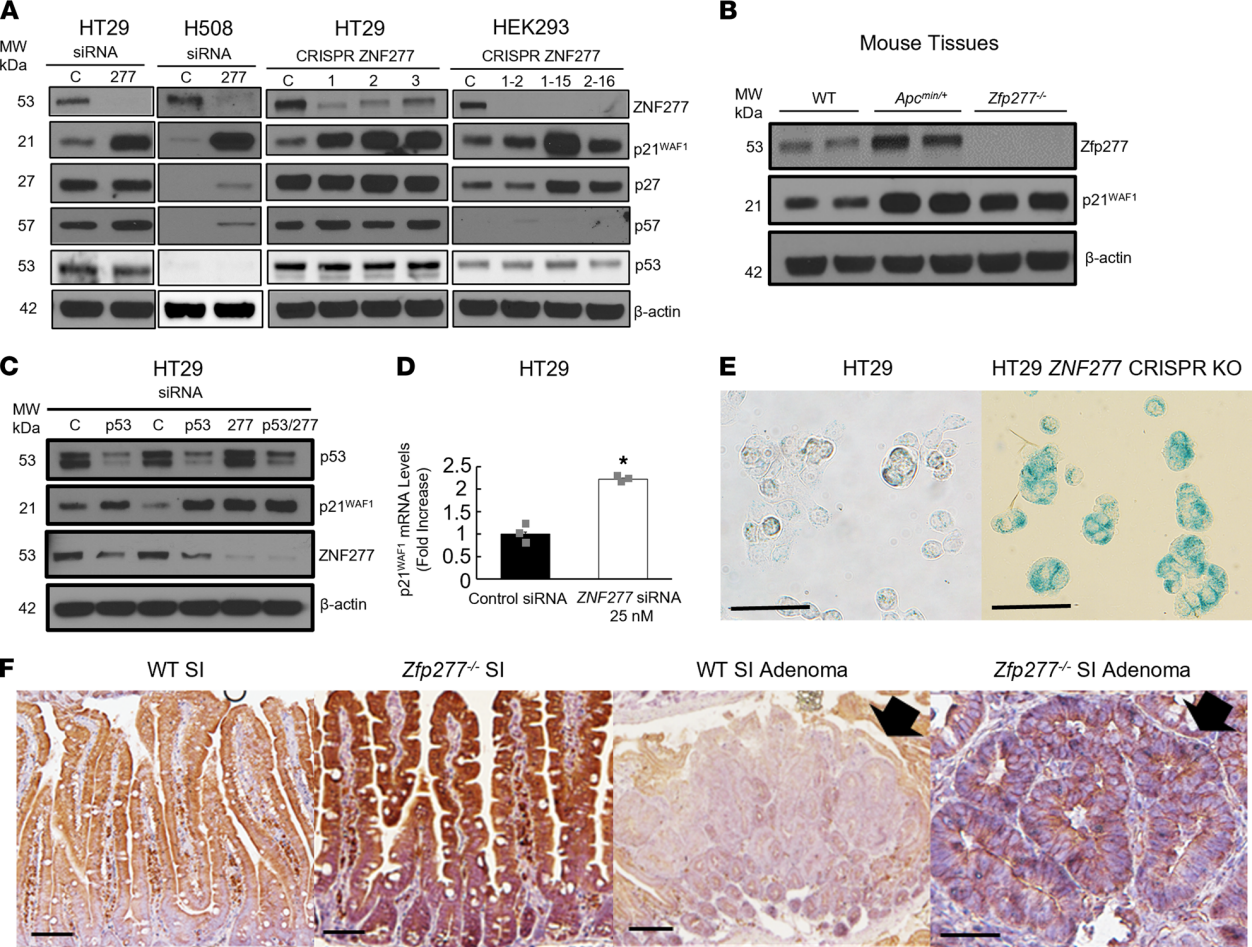
ZNF277 deficiency augments p21^WAF1^ expression. (**A**) siRNA and CRISPR knockdown of *ZNF277* expression augments p21^WAF1^ levels in HT29, H508, and HEK293 cells. β-Actin was used as a loading control. (**B**) Levels of murine p21^WAF1^ expression are augmented in colon tissue extracts from *Apc^Min/+^* and *Zfp277^–/–^* mice. Experiments were performed using tissues from 2 separate 8-week-old mice of each genotype. (**C**) Upregulated p21^WAF1^ expression after p53 and *ZNF277* knockdown in HT29 cells. All siRNAs were 25 nM, except lane 4 (50 nM). (**D**) *ZNF277* knockdown augments p21^WAF1^ mRNA levels in HT29 cells. Data are shown as mean ± SEM from 3 separate experiments. *P < 0.01 (2-tailed Student’s *t* test). (**E**) Zfp277 deficiency stimulates cellular senescence. β-Galactosidase staining in control HT29 cells (**A**) and HT29 cells with CRISPR knockdown of ZNF277 (**B**). Scale bar: 50 μM. (**F**) *Zfp277* deficiency increases p21^WAF1^ expression. IHC of p21^WAF1^ in the normal small intestine of WT (**A**) and *Zfp277^–/–^* (**B**) mice, as well as in small intestine adenomas from *Zfp277^+/+^Apc^Min/+^* (**C**) and *Zfp277^–/–^Apc^Min/+^* (**D**) mice. Arrows indicate adenomas. Scale bar: 100 μM.

Zfp277 regulates mouse embryonic fibroblast senescence (21). To gain additional insights into the role of *ZNF277* in modulating cellular senescence, we examined the effect of *ZNF277* CRISPR KO in colon cancer cells on markers for the senescence-associated secretory phenotype. As shown in Figure 7A, using *ZNF277* CRISPR gRNA constructs, we generated several *ZNF277* CRISPR KO cell lines in HT29 (pools) and HEK293 cells (clones). Compared with control cell pools derived from nonspecific (scrambled) gRNA, this greatly reduced or completely abolished ZNF277 protein expression.

In human HT29 and H508 colon cancer cells and HEK293 cells with siRNA- or CRISPR-induced reduction of *ZNF277* expression, we detected strikingly increased p21^WAF1^ levels (Figure 7A). Compared with WT mice, as shown in Figure 7B, we also detected robustly increased murine p21^WAF1^ expression in the colons of both *Apc^Min/+^* and *Zfp277*-deficient mice, whereas p27 levels were not affected. In HT29 cells with reduced *ZNF277* expression, we detected no changes in the expression of p27 or p57, other members of the p21^WAF1^ family (Figure 7A); we also detected no changes in p16^INK4A^ or p14^ARF^ expression (not shown). In HT29 cells, ZNF277 knockdown induced more than a 2-fold increased *p21^WAF1^* mRNA levels (Figure 7D). In addition, as shown in Figure 7C, p53 knockdown in HT29 cells increased p21^WAF1^ expression and did not alter the upregulation of p21^WAF1^ observed after *ZNF277* knockdown. These results suggest that, in colon cancer, *ZNF277* selectively regulates p21^WAF1^ expression independently of p53.

As anticipated from the above findings, CRISPR KO of *ZNF277* in HT29 cells increased β-galactosidase staining, a marker of augmented senescence (Figure 7E). As shown in Figure 7F, compared with the small intestine of *Apc^+/+^* mice and adenomas from *Apc^Min/+^* mice, we detected increased p21^Waf1^ expression in the small intestine of *Zfp277^–/–^* mice and in adenomas from *Apc^Min/+^ Zfp277^–/–^* mice. Collectively, these results suggest that *ZNF*2*77/Zfp277* represses p21^WAF1^ expression, at least partially via transcriptional regulation, to inhibit senescence. Our findings are consistent with observations by el-Deiry et al. that human intestines express p21^WAF1^ only in nonproliferating epithelial cells (i.e., differentiated enterocytes with absent *ZNF277* expression) but not in TACs that express *ZNF277* (34).

### RNA-Seq reveals ZNF277/Zfp277 transcriptional targets.

**Figure 8:**
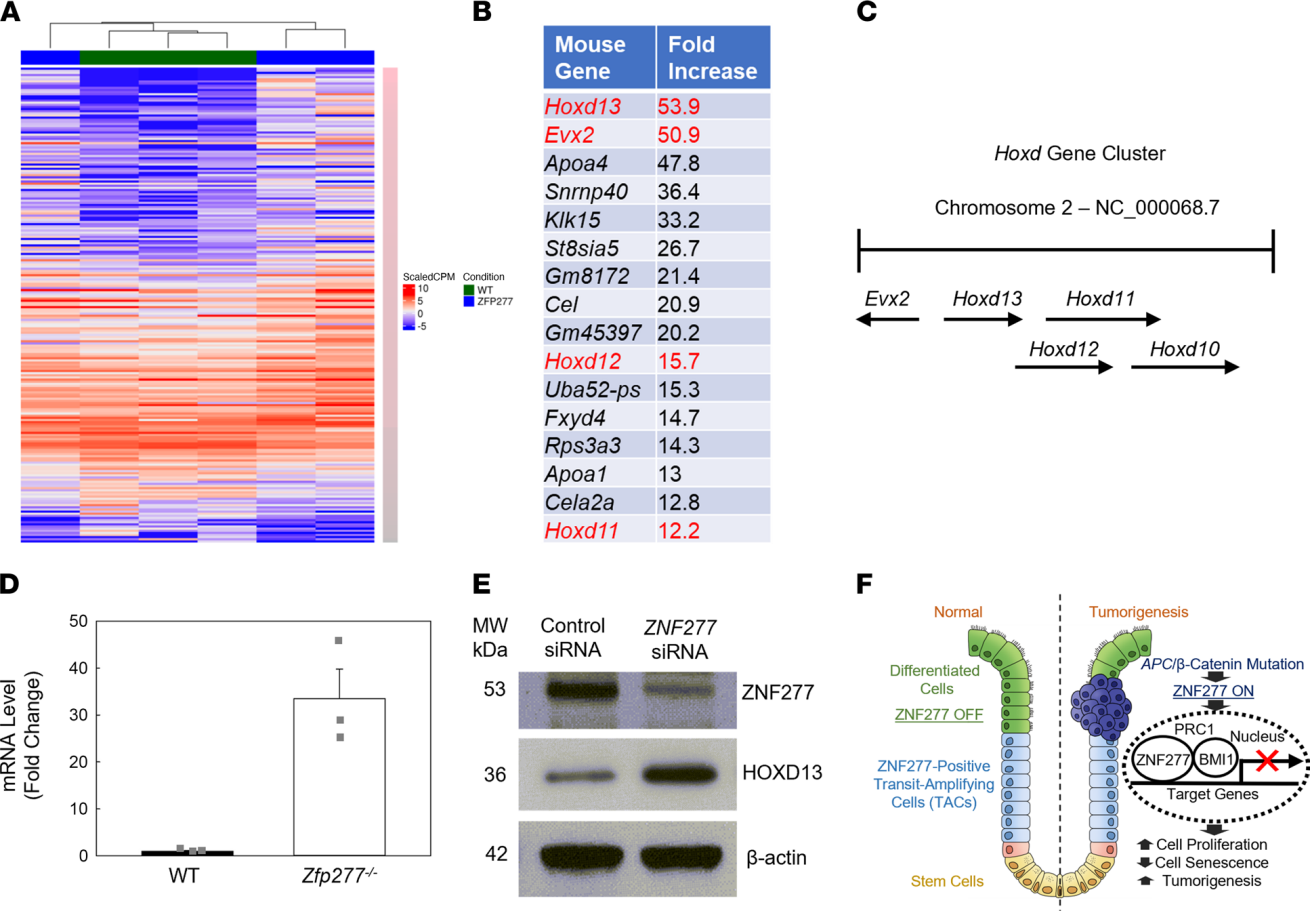
Zfp277 target genes in murine colon. Posterior Hoxd genes are Zfp277 transcriptional targets. (**A**) Heatmap of differentially expressed genes in colon mucosa from 3 WT mice and 3 *Zfp277^–/–^* littermates. (**B**) Top 16 upregulated genes from RNA-Seq, excluding immunoglobulins. Genes in the Hoxd cluster are highlighted in red. (**C**) Schematic of the murine *Hoxd* posterior gene clusters. (**D**) qPCR of *Hoxd13* mRNA expression in colon mucosal tissue from WT and *Zfp277^–/–^* mice. Values represent mean ± SEM (*n* = 3 each). (**E**) ZNF277 represses *HOXD13* gene expression. HOXD13 immunoblot of proteins extracted from ZNF277 CRISPR cell- and control cell–derived xenograft tumors (2 separate tumors from each group). (**F**) Model illustrating the role of *ZNF*2*77/Zfp277* in intestinal tumorigenesis. *ZNF277*, normally expressed in TACs but not in differentiated enterocytes, maintains intestinal homeostasis. Aberrant WNT signaling stimulates *ZNF277* overexpression in TACs. ZNF277 interacts with BMI1 in the PRC1 complex and represses p21^WAF1^ expression, thereby stimulating cell proliferation and attenuating cell senescence, as well as enhancing tumorigenesis and progressive neoplasia.

To identify murine Zfp277 transcriptional target genes, we performed RNA-Seq using RNA isolated from normal colonic mucosa from 8-week-old WT and *Zfp277^–/–^* male littermate mice (*n* = 3 for both). As shown by the heatmap in Figure 8A, based on a FDR cutoff of 0.05 and a log_2_ fold change (LFC) ≥ ± 1, in *Zfp277*-deficient versus WT mice, 758 genes were upregulated and 263 downregulated. Several homeobox genes belonging to the posterior *Hoxd* gene cluster (Figure 8, B and C) were dramatically increased, including *Hoxd13*, *EVX2*, *Hoxd12*, *Hoxd11*, and *Hoxd10* (all greater than 6-fold). Of the top 16 upregulated nonimmunoglobulin genes, 4 belonged to the posterior *Hoxd* gene cluster. Because read counts showed that the *Hoxd13* transcript was more abundant than the other 3 *Hoxd* cluster genes, we chose *Hoxd13* for more detailed characterization. As shown in Figure 8D, using qPCR, we observed more than a 30-fold increase in *Hoxd13* mRNA levels in colonic mucosa from *Zfp277*-deficient mice, confirming the RNA-Seq results. Consistent with these results, we detected increased HOXD13 protein expression in *ZNF277*-deficient xenografts (Figure 8E). Collectively, these results identify posterior *Hoxd* genes as *ZNF*2*77/Zfp277* transcriptional targets that are normally repressed in colon epithelium.

As shown in Supplemental Table 3, gene pathway analysis using DAVID 6.8 identified several enriched functionally related gene groups, including anterior-posterior pattern specification, pathway in cancer, regulation of MAPK, ERK1/2 cascades, canonical Wnt signaling, and cytokine-cytokine receptor interaction involved in immune system response/process. These findings suggest that *ZNF*2*77/Zfp277* has a broad range of transcriptional target genes and may play important roles at different stages of development and cancer progression. The observation that murine *Zfp277*-deficient mice are healthy and fertile (21) suggests that other transcription factors may replace Zfp277 function when it is absent.

To identify human ZNF277 target genes in cancer cells, we performed RNA-Seq using RNA isolated form HT29 cells with and without ZNF277 CRISPR KO (*n* = 3 for both). As shown in Supplemental Table 4, based on a FDR cutoff of 0.05 and a LFC ≥ ± 1, in ZNF277-deficient compared with control HT29 cells, 657 genes were upregulated and 93 were downregulated. The top 4 altered KEGG signaling pathways in cancer included increased expression of p21^WAF1^, pathways regulating pluripotency in stem cells, proteoglycans, and PI3K/AKT signaling. These findings provide additional evidence that ZNF277 plays a prominent role in human CRC by transcriptional regulation of many genes involved in cancer progression, including p21^WAF1^ and Wnt signaling. Consistent with our Zfp277 RNA-Seq findings, many HOX genes were identified as ZNF277 targets, revealing Hox genes as transcriptional targets of Zfp277, most likely playing a role in early development.

## Discussion

Intestinal epithelial homeostasis is maintained by intestinal stem and early progenitor TACs (35, 36). Stem cells can divide asymmetrically and generate 2 daughter cells, a stem cell and pluripotent TAC. TACs proliferate rapidly to generate differentiated cell types, including goblet cells and functional enterocytes, thus playing a critical role in directing stem cell activity toward cell proliferation and differentiation. TAC dysregulation disrupts intestinal homeostasis, and TAC accumulation drives tumorigenesis (37). The delicate balance between stem cells and TACs is maintained by several genetic mechanisms, including chromatin remodeling and transcriptional, posttranscriptional, and translational modifications (35, 36). Underlying these mechanisms is the expression by TACs of a unique set of genes, including the cell proliferation marker Ki67 and protooncogene *MYC*. *MYC*, a prominent oncogene and transcription factor, is also expressed primarily in TACs, plays an essential role in early embryonic development, and acts as a protooncogene in tumor cells (38). *Myc*, the mouse homologue of human *MYC*, is expressed only in intestinal TACs and is required for intestinal crypt formation but dispensable for epithelial homeostasis (39). A *Myc-*null mutation results in embryonic lethality, whereas *Apc^Min/+^* mice in which *Myc* is haploinsufficient survive longer than control littermates due to delayed intestinal adenoma formation (40).

Our findings support a key role for ZNF277, an evolutionarily conserved zinc finger transcription factor, in cell senescence and oncogenesis. We identified ZNF277/Zfp277 as a potentially novel TAC-specific transcription factor that promotes TAC proliferation and intestinal tumorigenesis, thereby shedding new light on the regulation of TACs, a crucial link between intestinal stem cells and differentiated enterocytes. Indeed, the expression pattern and functions of *ZNF277* appear to closely mimic those of *MYC*. The mouse ENCODE transcriptome database (NCBI Gene; https://www.ncbi.nlm.nih.gov/) and Human Protein Atlas (27) reveal *ZNF*2*77/Zfp277* mRNA and protein expression in a wide variety of normal murine and human tissues, including the intestines, with strong nuclear staining in 22 of 24 human colon tumors. Previously, using 12 archived human colon cancer tissues, we detected *ZNF277* mRNA overexpression in cancer compared with adjacent normal colon mucosa (17). Likewise, in an archived set of 23 formalin-fixed paraffin-embedded human colon cancer tissues, we used IHC to detect ZNF277 protein overexpression in tumors compared with adjacent normal colon from the same person; ZNF277 was primarily localized to tumor cell nuclei (17). These findings are supported by in silico analysis of the NCBI Gene Expression Omnibus (GEO) profile database that revealed upregulated *ZNF277* expression in 33 of 34 colon tumors (97%) compared with adjacent normal colon (17, 41).

In the present study, analysis of *ZNF277* expression using 4 online cancer databases revealed that both *ZNF277* transcript and protein levels were increased in CRC specimens from men and women of all ages and races, with a variety of cancer stages and lymph node metastasis. Per cBioPortal, *ZNF277* gene mutations causing mostly missense changes were identified in approximately 2.5% of more than 3000 colon cancer specimens. Because we identified *ZNF277* as a potential colon cancer oncogene, it is not surprising that its rates of mutation in colon cancer (mostly loss of function) are not as high as reported for tumor suppressors like *APC* (42) and *p53* (43). Indeed, our studies in a murine model of genetic colon cancer, *Apc^Min/+^* mice, supports an important role for *ZNF*2*77/Zfp277* in intestinal neoplasia. *Zfp277* deficiency profoundly reduced tumor formation in both the small intestine and colon of *Apc^Min/+^* mice. Reduced tumor formation was associated with strikingly prolonged survival in both male and female mice, suggesting that ZNF277 may be an important CRC oncogene.

The polycomb proteins, polycomb repressive complex 1 and 2 (PRC1 and PRC2), play pivotal roles in stem cell fate determination and development, primarily by maintaining the repressed state of target genes via histone modifications (44–47). PRCs are important gatekeepers that establish and maintain cell identity. In addition to pluripotent embryonic stem cells, PRCs and associated proteins — such as BMI1, a polycomb ring finger protein — also function in tissue-specific stem cells. PRC1 preserves intestinal stem cell identity by suppressing non-lineage-specific transcription factors, thereby sustaining Wnt/β-catenin transcriptional activity (48). In mouse embryonic fibroblasts, Negishi et al. showed that Zfp277 mediates transcriptional repression of p16^Ink4A^ and p19^ARF^ via interaction with Bmi1 in the PRC1 complex (21). Loss of Zfp277 in mouse embryonic fibroblasts caused dissociation of PRC1 proteins from the Ink4A/ARF locus, resulting in premature senescence associated with derepressed p16^Ink4A^ and p19^ARF^ expression (21). Liu et al. showed that mouse embryonic fibroblasts with strong p16^Ink4A^ promoter activation in vivo display features of senescence (49). The INK/ARF locus, which generates tumor suppressors p16^INK4A^ and p15^INK4B^, is a pivotal node between senescence and cancer (23, 24). p16^INK4A^ and p15^INK4B^ bind to CDK4/6 to induce cell cycle arrest via retinoblastoma protein, whereas p14^ARF^/19^ARF^ arrests the cell cycle by stabilizing p53, trapping MDM2, and increasing expression of p21^WAF1^, an important cell cycle inhibitor and tumor suppressor for many cancers including those of the colon. Here, we showed that ZNF277 also interacts with BMI1 in human colon cancer cells, although the functional significance of this interaction and whether ZNF277 is a component of the PRC1 complex in intestinal epithelial cells remains to be determined.

We found that *ZNF277* inhibits cellular senescence by repressing p21^WAF1^ expression in human colon cancer cells. p21^WAF1^ is a potent cell cycle inhibitor and tumor suppressor (50, 51) whose expression is lost in ~80% of colon cancers (52); p21^WAF1^ loss correlates with a poor prognosis (50). p21^WAF1^-deficient mice develop spontaneous intestinal tumors (53), and in human fibrosarcoma cells, p21^WAF1^ overexpression induces growth arrest and senescence by inhibiting cell cycle progression and DNA repair (54). p21^WAF1^ is also a critical determinant of intestinal cell responses to the nonsteroidal antiinflammatory drug sulindac; in *Apc^Min/+^* mice, inactivating p21^WAF1^ eliminates the ability of sulindac to inhibit intestinal tumor formation (55). Many FDA-approved anticancer drugs, including histone deacetylase inhibitors, function at least in part by inducing p21^WAF1^ expression. Campaner et al. showed in various cell types, including MEFs and a p53-null human cancer cell line, that p21^WAF1^ suppresses cellular senescence induced by MYC activation (56). Here, we found that ZNF277 inhibits p21^WAF1^ expression in human CRC cells by a p53-independent mechanism. These findings are also consistent with our finding that increased *ZNF277* levels in CRC correlate significantly with reduced p21^WAF1^ (*CDKN1A*) expression. Whether ZNF277-mediated suppression of p21^WAF1^ expression depends on PRC1 remains to be determined.

Expecting to identify a broad range of Zfp277 transcriptional targets, including genes important for development, we used RNA-Seq to examine the effects of *Zfp277* deficiency in murine colonic mucosa. This approach identified murine *Hoxd* gene clusters as *Zfp277* targets. In mammals, 39 *HOX* genes play critical roles in development (57). These evolutionarily conserved genes are clustered at 4 distinct loci, *HOXA* to *HOXD* (57). The posterior *HOXD* genes — *HOXD10*, *HOXD11*, *HOXD12*, and *HOXD13* — primarily establish posterior expression boundaries in limb and gut development. Mutant mice with spontaneous deletions that remove the entire posterior *Hoxd* cluster genes are viable but have hindlimb paralysis (58), whereas a nonsense mutation of human *Hoxd13* causes synpolydactyly (59). Roberts et al. show that misexpression of Hoxd13 in the primitive midgut mesoderm is sufficient to transform the midgut into a structure resembling the hindgut (60). In addition to regulating anterior posterior limb development and morphogenesis, these *Hox* genes also play important roles in cellular identity, cell proliferation, stem cell differentiation, gut growth and maturation, and gastrointestinal cancer (61–64). Many homeobox-containing transcription factors modulate cell proliferation by regulating cell cycle proteins, including p21^WAF1^, either at the transcriptional level or via protein-protein interactions (65). Our work reveals an intriguing link between the role of Zfp277 in early development and cancer. After embryogenesis, human posterior *HOXD* genes are only expressed in a few organs, including the colon (27). This restrictive expression pattern suggests a potentially important role for posterior *HOXD* genes in colon pathology. The role of posterior *HOXD* genes in colon cancer is not well defined; *HOXD8* and *HOXD12* expression are reduced in colon cancer (66), whereas *HOXD10* expression may be increased (67). Interestingly, posterior *HOXD* genes are repressed by polycomb group proteins (PcG) proteins including PRC1 (68). The functional significance of this ZNF277/HOXD axis in CRC progression remains to be determined. Likewise, the role of Zfp277 transcriptional regulation of other pathways involved in cell proliferation and cancer progression, including MAPK/ERK signaling, canonical Wnt signaling, and cytokine–cytokine receptor interaction, will be the focus of future studies.

To define how silencing ZNF277 affects transcriptomes related to proliferation in human CRC cells and to identify potential ZNF277 target genes in CRC, we performed RNA-Seq in HT29 cells with and without ZNF277 deficiency. The leading pathways impacted by ZNF277 deficiency included increased p21WAF1 expression in cancer stem cell signaling, proteoglycans in cancer, and PI3K/AKT signaling. These findings reveal that ZNF277 may regulate CRC progression via a variety of molecular mechanisms involving p21^WAF1^, intestinal stem cells, and Wnt/β-catenin and other cancer signaling pathways. Notably, HOX genes were again identified and confirmed the key role of ZNF/Zfp277 in development and cancer progression. A limitation of an RNA-Seq–based approach is that genes identified by RNA-Seq differential gene expression may not be direct transcriptional targets of Zfp277. This limitation will be overcome when ChIP quality anti-ZNF/Zfp277 antibodies become available for direct ChIP-Seq.

Wnt/β-catenin signaling is essential for normal intestinal homeostasis. Hence, the major Wnt signaling components and key downstream targets such as *APC*, *CTNNB1*, and *MYC* are challenging therapeutic targets. In contrast, several observations suggest that ZNF277 may be a more promising and druggable target. First, Zfp277-deficient mice are healthy (21), suggesting a limited risk of off-target toxicity. Second, the interaction between ZNF277 and BMI1 in the PRC1 complex provides a useful screening tool to test potential small molecule inhibitors of ZNF277. Lastly, inhibiting ZNF277 may increase p21^WAF1^ expression and promote cell cycle arrest in cancer cells. As summarized by the illustration in Figure 8F, the present work newly identifies *ZNF*2*77/Zfp277* as an intestinal TAC marker and colon cancer oncogene. ZNF277 modulates intestinal β-catenin signaling and tumorigenesis by acting as a key transcription factor and component of the PRC1 complex that regulates cell proliferation and senescence. It would be of interest to determine whether the oncogenic effects of ZNF277/Zfp277 depend solely on its expression in intestinal epithelial cells; this question will be addressed in future studies by examining the effects on intestinal tumorigenesis in mice with conditional intestinal epithelial cell–selective *Zfp277* deficiency.

## Methods

### Chemicals

Chemicals were purchased from Sigma-Aldrich and cell culture media were purchased from Thermo Fisher Scientific.

### Online cancer databases

#### Oncomine database and platform.

Oncomine was an online cancer microarray database and an integrated data-mining platform. *ZNF277* mRNA levels in colon cancer tumor specimens were compared with normal surrounding tissues. The threshold and threshold *P* value used were 1.5-fold and 0.001, respectively. 

#### GEPIA server.

GEPIA (gepia.cancer-pku.cn) is an interactive web server for analyzing RNA-Seq expression data using tumor and normal samples from the National Cancer Institute Cancer Genome Atlas Program (TCGA) database and the Genotype-Tissue Expression (GTEx) project (https://www.genome.gov/Funded-Programs-Projects/Genotype-Tissue-Expression-Project). It provides customizable functions according to cancer types, including differential gene expression. The Colon Adenocarcinoma (COAD) data sets were used in our analysis of ZNF277 transcript.

#### The Human Protein Atlas program.

This online database (https://www.proteinatlas.org/) provides information on human proteins in cells, tissues, and organs using integration of various omics technologies, including antibody-based imaging, mass spectrometry–based proteomics, transcriptomics, and systems biology.

#### UALCAN server.

UALCAN (ualcan.path.uab.edu) is an interactive web resource for analyzing cancer OMICS data, and it provides easy access to OMICS data (TCGA and MET500). The TCGA COAD data set was used in our analysis of *ZNF277* transcript.

#### Experimental animals.

Six- to 8-week-old WT C57BL/6J and *Apc^Min/+^* (C57BL/6J-*Apc^Min^*/J) mice were purchased from The Jackson Laboratory. *Zfp277*-KO mice (21) (B6.129-Zfp277<tm1>) were purchased from the Riken BioResource Research Center. We generated WT, *Apc^Min/+^* *Zfp277^+/–^,* and *Apc^Min/+^* *Zfp277^–/–^* mice by breeding *Apc^Min/+^* *Zfp277^+/–^* male mice with *Zfp277^+/–^* female mice. Briefly, we first crossed male *Apc^Min/+^* (C57BL/6J-*Apc^Min^*/J) mice (The Jackson Laboratory, stock no. 002020) with female *Zfp277^+/–^* mice and then crossed *Apc^Min/+^ Zfp277^+/–^* male mice with *Zfp277^+/–^* female mice to generate *Apc^Min/+^ Zfp277^+/+^*, *Apc^Min/+^ Zfp277^+/–^*, and *Apc^Min/+^ Zfp277^–/–^* mice. Genotyping for *Zfp277* and *Apc^Min^* status was performed using tail genomic DNA per instructions from the Riken BioResource Research Center and The Jackson Laboratory, respectively (15–17, 21), and Zfp277 protein deficiency was confirmed by immunoblotting. Using different anti-Zfp277 antibodies directed against various portions of the Zfp277 protein, including the N-terminus, we failed to detect Zfp277 protein in selected tissues from *Zfp277^–/–^* mice including liver (Supplemental Figure 3A) and colon (Figure 6C). At 15 weeks of age, mice were euthanized, the intestines were harvested, and tumors were counted as previously described (15–17, 69). After counting tumors, tissues were fixed in formalin and stained for H&E in Swiss Roll configurations (Supplemental Figure 3, B–D). For experiments involving immunoblotting and qPCR, we used 6- to 8-week-old mice. To characterize intestinal tumors, we used 15-week-old mice. We housed mice under identical conditions in the same pathogen-free room with free access to commercial rodent chow and water; we allowed mice to acclimatize in the vivarium for 2 weeks prior to breeding. For survival studies, mice were observed daily and euthanized when moribund or achieving predetermined endpoints with at least 4 of the following signs of severe distress: dehydration, rectal prolapse or bleeding, anorexia, weight loss >20% initial body weight, hunched posture, lethargy, persistent recumbency, dyspnea, ruffled fur, and inability to rise or ambulate.

#### Hematocrit measurements.

At the age 15 weeks, blood was obtained from 3 male and 3 female mice by cardiac puncture after mice were anesthetized preceding euthanasia as described (70). After centrifuging heparin-coated microhematocrit tubes at 12,000*g* for 30 seconds at room temperature, the ratio of the volume occupied by RBCs to the volume of whole blood was measured and expressed as a fraction in percentage.

#### Tumor measurement, histological, and IHC analyses.

At age 15 weeks, experimental mice were weighed and euthanized. The small intestine and colon were harvested, and segments were opened longitudinally and placed flat with the luminal surface facing up on transparent films. Tumors were identified by visual inspection and counted using a Nikon SMZ1500 dissecting microscope by investigators masked to genotypes and experimental groups. Colon tumor sizes were measured using calipers. For histological analysis, tissues were fixed in 4% paraformaldehyde and paraffin-embedded in Swiss Roll configurations (Supplemental Figure 3D). Sections (5 μm) were stained with H&E (Supplemental Figure 3B). For IHC and immunofluorescent analysis, we used primary and secondary antibodies listed in Supplemental Table 1, including names of manufacturers, antibody catalog numbers, and lot numbers. Immunoglobulins and secondary antibodies were used as negative controls.

#### In vivo and in vitro measurement of cell proliferation and apoptosis.

Active proliferating intestinal epithelial cells were labeled with BrdU 2 hours before euthanasia (15). Briefly, to label S-phase cells, 2 hours before euthanasia, mice were administered an i.p. injection of 50 mg/kg BrdU (Sigma-Aldrich), a marker of cell proliferation. The percentage of BrdU^+^ cells was determined after immunostaining with anti-BrdU antibody (Supplemental Table 1). We also performed Ki67 IHC staining to measure actively proliferating cells; we counted Ki67^+^ nuclei in intestinal crypts (data expressed as percentage of total cells that were Ki67^+^ in each crypt). A total of 10 random crypts per mouse were examined in tissue sections from 3 mice of each genotype. To identify apoptotic cells, we immunostained tissue sections with anti–activated caspase-3 antibody (Supplemental Table 1). Only complete crypts were evaluated, and investigators were masked to genotype and experimental groups.

#### Murine enteroid cultures.

Small intestinal enteroids were generated from primary tissues using the mouse Intesticult Organoid Growth Medium (Stem Cell Technologies [SCT]; catalog 06005) and Corning Matrigel Matrix (Corning Inc.; catalog 356231) per SCT protocol. Enteroids were maintained with media changes every 2–3 days and passaging when growth reached appropriate densities. ROCK 1/2 inhibitor (Y-27632) at 10 μM was added for the first 2 days after generation from primary tissue.

#### Cell lines and cell culture.

HT29, H508, and SNUC4 human colon cancer cells and HEK293 human kidney epithelial cells were purchased from the American Type Culture Collection (ATCC) and maintained in growth media supplemented with 10% FBS. HT29 and SNUC4 cells were grown in McCoy’s 5A Media (Thermo Fisher Scientific). H508 and HEK293 cells were grown in RPMI-1640 and Leibovitz’s L-15 media, respectively. We passaged adherent cells weekly at subconfluence after trypsinization and maintained cultures in incubators at 37°C in an atmosphere of 5% CO_2_ and 95% air. All cell lines were authenticated a minimum of every 6 months by short tandem repeat sequencing in the UMB Genomic Core.

#### Plasmids and transient transfection.

Transient ZNF277 overexpression in human HT29 colon cancer cells was achieved by transfecting 1 μg of plasmid pcDNA3.1-*ZNF277* (clone OHu03772; GenScript) for 48 hours. Nuclear lysates were then analyzed for ZNF277 levels using immunoblotting and compared with HT29 cells transfected with pcDNA3.1.

#### Measurement of in vitro cell proliferation.

Cells were seeded in 96-well plates at approximately 10% confluence and allowed to attach for 24 hours. After an additional 24 hours, cell proliferation was determined by adding 20 μL CellTiter 96 AQueous One solution (Promega) to each well. After a 1- to 2-hour incubation at 37°C, absorbance was measured at 490 nm using a 96-well microtiter plate reader (SpectraMax384).

#### siRNA transfection.

We purchased the following siRNAs from Invitrogen: Silencer Select Negative Control No. 1 siRNA, *ZNF277* SILEE SELECT SIRNA ASSAY ID S22065, and CTNNB1 SILEER SIRNA ASSAY ID 146154. For siRNA transfection experiments, human colon cancer cells and HEK293 cells were seeded in 6- and 96-well plates at approximately 10% confluence and incubated at 37^o^C for 24 hours. siRNA duplex oligos targeting *ZNF277* or nontargeting control oligos were transfected into cells with Lipofectamine Transfection Agent (Thermo Fisher Scientific) according to the manufacturer’s instructions. One to 2 days following transfections, cells in 96-well plates were used for cell proliferation assay, and cells in the 6-well plates were harvested for immunoblotting to confirm and quantify siRNA knockdown.

#### Generation of CRISPR KO cell lines.

We purchased 2 *ZNF277* CRISPR gRNA plasmids (project name U4370DK190-1; clone C93266, gRNA TTGCAGTTTACAATGTTGTC; project name U4370DK190-2, clone C93269, gRNA AGACAGTAAGCATTGTATCC) from GenScript. The cloning vector for both gRNAs is the pS.pCas9 BB-2A-Puro (PX459) v2.0 plasmid. After verifying these gRNA plasmid sequences by DNA-Seq, we used SuperFect transfection reagent (QIAGEN, catalog 30130S) to transfect HT29 and HEK 293 cells and used HT29 and HEK 293 cells transfected with the pS.pCas9 BB-2A-Puro vector as controls. Stably transfected cells were selected using 10 μg/mL puromycin based on killing curves. Finally, we used immunoblotting to determine ZNF277 protein levels. Using the protocol above, we generated several pools of mixed *ZNF277* CRISPR cells with low or absent ZNF277 expression in HT29 and HEK 293 cells.

#### Generation of human colon cancer xenografts.

To generate xenografts, 1 million pooled CRISPR *ZNF277*-KO HT29 cells (pool no. 1) or nonspecific CRISPR HT29 cells were injected s.c. into both flanks of 6-week-old female nude mice (stock no. 002019; Homozygous for Foxn1^nu^; The Jackson Laboratory) in 100 μL mixtures (50% Matrigel) as previously described (71). *ZNF277* CRISPR HT29 cells were injected into the left flanks of nude mice, whereas the nonspecific CRISPR HT29 cells were injected into the right flanks of the same nude mouse. Tumor size was measured biweekly with calipers and tumor volumes calculated using the formula: tumor volume = (length × width^2^)/2. Mouse body weight was measured twice per week. At the end of the study, xenografts were excised, photographed, and weighed. Tumors were bisected and half stored in liquid nitrogen for molecular studies and half fixed for IHC studies.

#### Antibodies and immunoblotting.

Immunoblotting were performed as described previously (72). To ensure equal loading of protein samples, protein concentrations of cell lysates were determined using the BCA Protein Assay kit (catalog 23227; Thermo Fisher Scientific). Protein (10–20 μg) was loaded into each lane. Antibodies used in immunoblotting, including manufacturers, catalogs, and lot numbers, are listed in Supplemental Table 1. After probing with primary antibodies, immunoblots were incubated with horseradish peroxide–conjugated secondary antibodies and visualized by chemiluminescence (Pierce) using the ChemiDoc Touch Imaging System (Bio-Rad). To avoid saturation areas of the bands and apply the linear ranges only, we performed semiquantifications using the Quantity One software (Bio-Rad).

#### IP.

IP was performed following New England Biolab protocols with minor modifications. Briefly, cells were lysed in RIPA buffer with proteinase and phosphatase inhibitors. Lysates were precleared with magnetic protein G agarose beads (New England Biolabs), incubated with 2 μg anti-BMI1 antibody (Supplemental Table 1) overnight. Normal mouse IgG (2 μg) was used as a negative control. Anti-ZNF277 and anti-BMI1 antibodies were used to detect ZNF277 and BMI1, respectively. IP was performed using actively growing cells with 10% FBS.

#### qPCR and RT-PCR.

We performed qPCR and quantification of mRNA levels as described previously (73). We confirmed the specificity of amplifications by melting-curve analysis and calculated relative levels of mRNA according to the standard ΔΔCt method. We normalized expression values by comparison with GAPDH. qPCR and RT-PCR primer sequences are listed in Supplemental Table 2.

#### ChIP assays.

We performed ChIP assays using the Pierce Magnetic ChIP Kit (catalog 26157; Thermo Fisher Scientific) per manufacturer’s instructions as previously described (74). Briefly, in vivo crosslinking was performed using 4 million cultured cells using 1% formaldehyde. Cell lysis was performed using buffers containing proteinase inhibitor cocktails. Lysates were then digested with Micrococcal Nuclease (MNase; Pierce Kit, Thermo Fisher Scientific) to generate random DNA fragments from 160 to 320 bp. Chromatin was obtained after brief sonication to rupture nuclei. Aliquots (5 μL) were removed for agarose gel analysis. IP of crosslinked protein/DNA were performed overnight at 4°C using antibodies against mouse β-catenin (Supplemental Table 1), RNA Polymerase II (positive control) and rabbit or goat IgG (negative controls). Elution and reverse crosslinks of protein/DNA complexes to free DNA were performed using the ChIP Elution Buffer without protein kinase K, using a magnet. Eluted DNA was purified using DNA spin columns. qPCR was performed with primers listed in Supplemental Table 2 using eluted DNA and 1% input. Antibodies used in this ChIP assay included mouse monoclonal anti–human β-catenin antibody, rabbit anti–RNA Polymerase II, and mouse IgG (Supplemental Table 1). GAPDH was used as a negative control.

#### RNA-Seq.

RNA-Seq was performed by the Genomic Resource Center (GRC) at the Institute for Genome Sciences at the University of Maryland Baltimore. Briefly, we provided high-quality total RNA and submitted the samples to GRC, which then conducted quality control tests, prepared Illumina RNA-Seq libraries, and performed sequencing using the Illumina HiSeq4000 System. Differentially expressed transcripts were identified based on a FDR cutoff of 0.05 and a LFC ≥ ±1. Gene pathway analysis was performed using DAVID Bioinformatics Resources 6.8 (David.ncifcrf.gov). For Zfp277 RNA-Seq, we used RNA isolated from normal colonic mucosa from 8-week-old WT and *Zfp277^–/–^* male littermate mice (*n* = 3 for both). Comparisons were made using WT (control no. 3). For ZNF277 RNA-Seq, we used RNA isolated from control HT29 cells and 3 HT29 cell lines with CRISPR KO (*n* = 3 for both; Figure 7A). Comparisons were made using control no. 1. The GEO accession for this work is GSE192559.

#### Statistics.

Data were expressed as mean ± SEM from a minimum of 3 independent experiments. We performed Student’s 2-tailed *t* test, Mann-Whitney *U* test, 1-way ANOVA, with either Tukey’s HSD post hoc or Dunn’s tests using SigmaPlot 13.0 (Systat Software Inc.) and considered *P* < 0.05 to be statistically significant.

#### Study approval.

For human samples, deidentified preexisting formalin-fixed paraffin-embedded sections from surgically resected human colon cancers and adjacent normal colon or small intestine tissues from the same individuals were obtained from the Department of Pathology at the University of Maryland Baltimore (an exemption for these studies was obtained from the IRB at the University of Maryland Baltimore). This study abides by the Declaration of Helsinki principles. The animal study was approved by the Office of Animal Welfare Assurance at the University of Maryland School of Medicine and by the Research and Development Committee at the VA Maryland Health Care System.

## Author contributions

ZP, JL, GX, and SML performed experiments. CBD and HY provided human tissues and expertise in pathology. GX performed online cancer database analysis. GX and JPR designed and supervised the study and wrote the manuscript. All authors reviewed, edited, and approved the submitted manuscript.

## Supplementary Material

Supplemental data

## Version 1 01/11/2022

In-Press Preview

## Version 2 02/22/2022

Electronic publication
