# Supplementary material for: Zinc finger protein 277 is an intestinal transit-amplifying cell marker and colon cancer oncogene
Source: JCI Insight. 2022 Feb 22;7(4):e150894. doi: 10.1172/jci.insight.150894 (PMC8876557; doi:10.1172/jci.insight.150894)
Supplement: Supplemental data [file jciinsight-7-150894-s220.pdf]

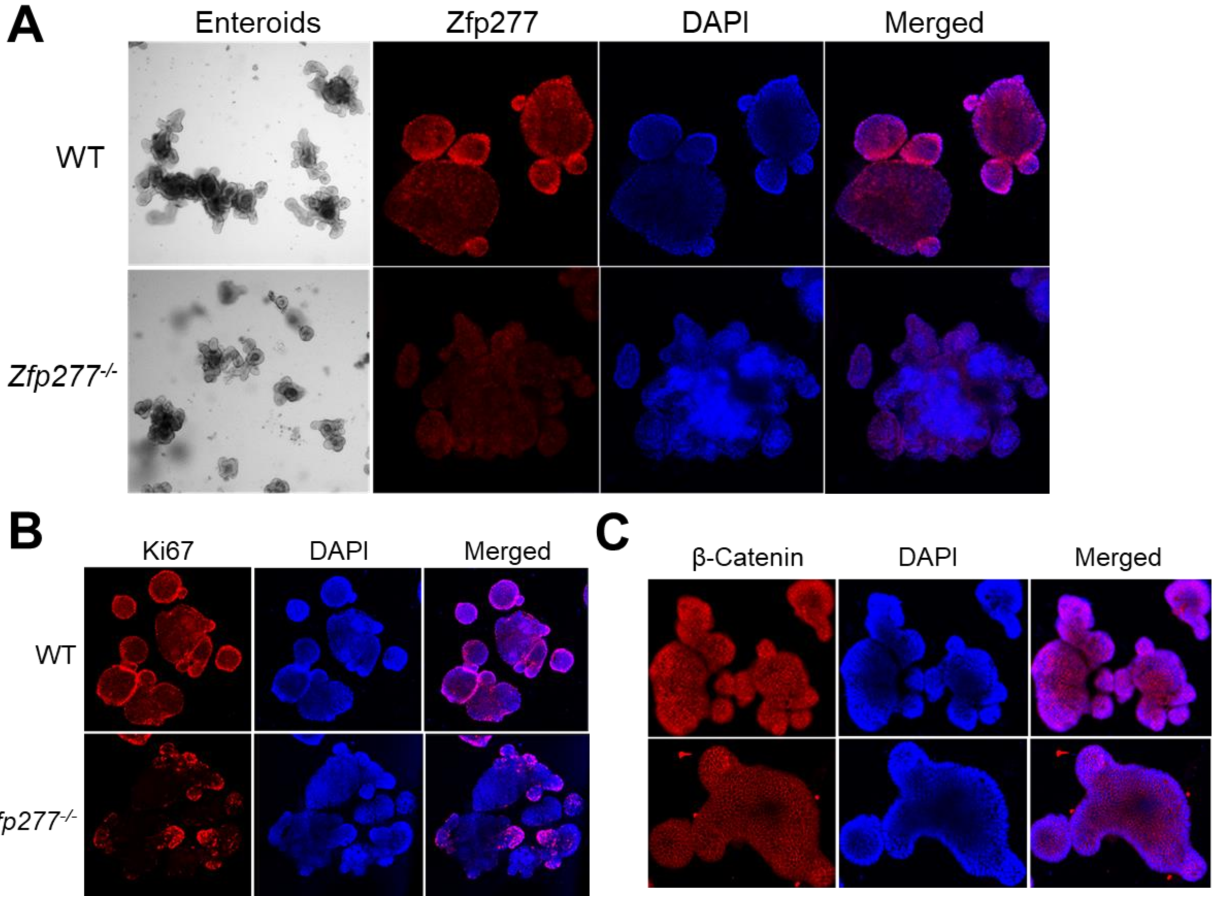

Supplemental Figure 1. Zfp277, Ki67 and β-catenin expression in enteroids cultivated from WT and *Zfp277$^{-/-}$* mice. (**A**) Representative light micrographs of enteroids, IF images of Zfp277, DAPI and merged confocal image of DAPI and Zfp277. (**B** and **C**) Representative confocal micrographs of enteroids stained with anti-Ki67 (B) and anti-β-catenin antibodies (C) and merged images. 10X.

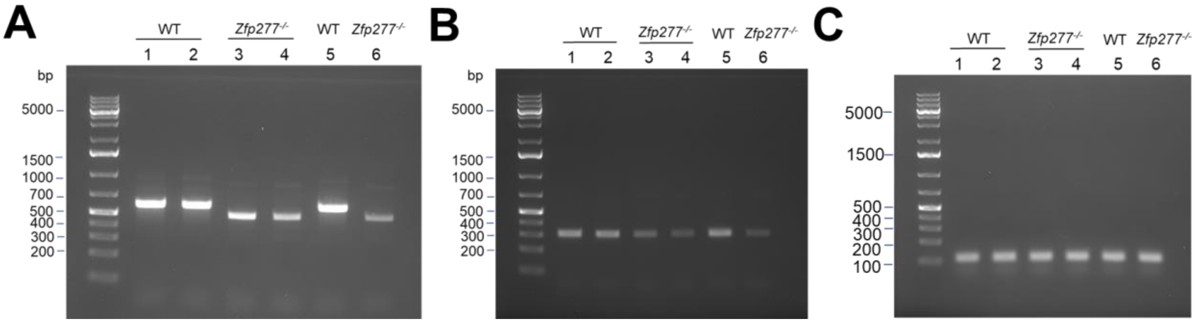

Supplemental Figure 2. *Zfp277* transcript variant 1 and 2 levels in murine colon mucosa and small intestine enteroids. **(A)** Variant 1 expression. Lanes 1 and 2, *Zfp277* mRNA RT-PCR product using cDNA prepared from colonic mucosa from two different WT mice. Lanes 3 and 4, *Zfp277* mRNA RT-PCR product using cDNA prepared from colon mucosa from two different *Zfp277*-deficient mice. Lane 5, *Zfp277* mRNA RT-PCR product using cDNA prepared from enteroids from WT mice. Lane 6, *Zfp277* mRNA RT-PCR product using cDNA prepared from enteroids from *Zfp277*-deficient mice. **(B)** Variant 2 expression. Lanes are the same as in A. **(C)**. *Gapdh* RT-PCR. Lanes are the same as in A and B.

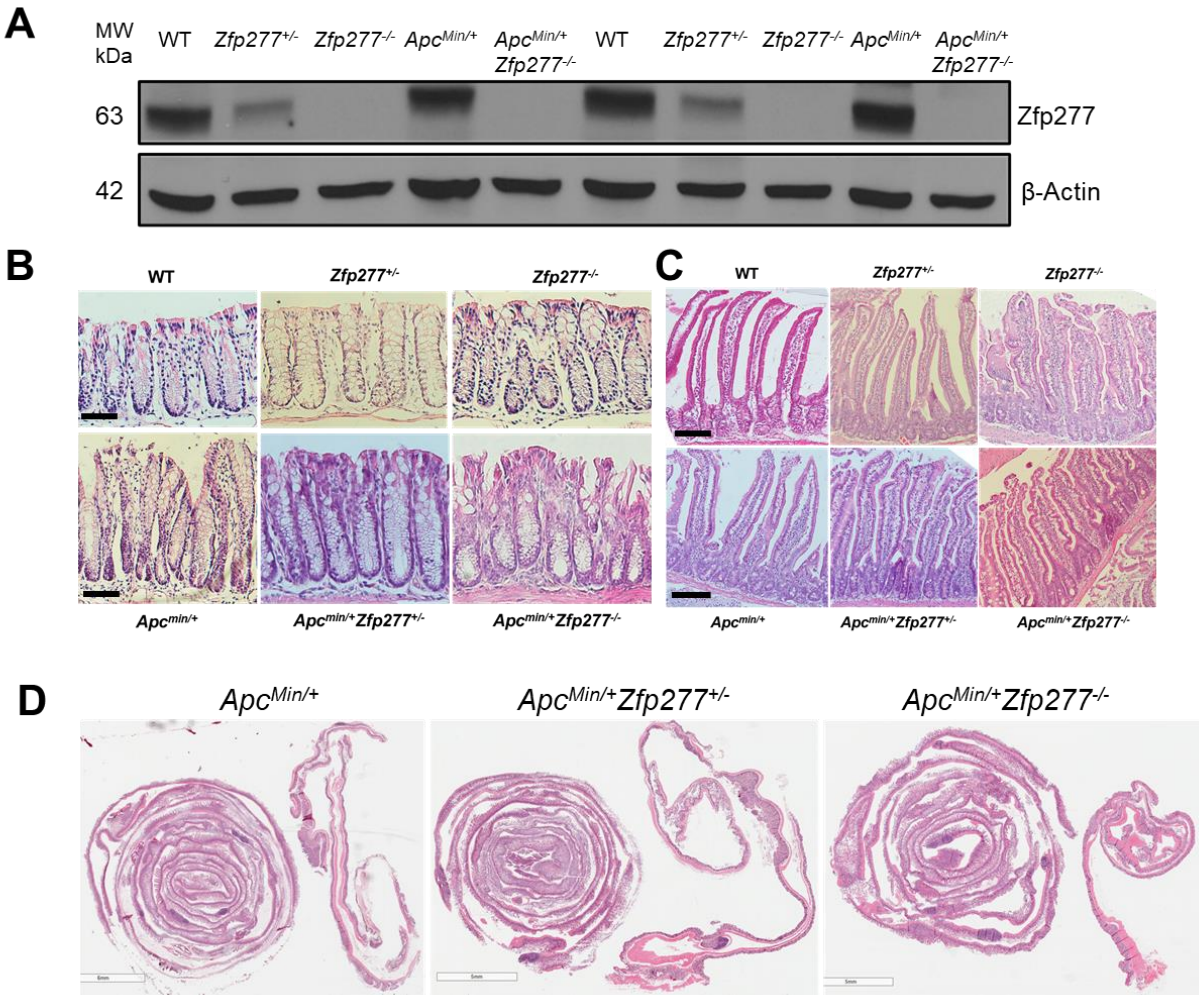

Supplemental Figure 3. **Characterization of *Zfp277* expression in transgenic mice.** (**A**) Representative Zfp277 immunoblots using liver tissue extracts from WT, $Zfp277^{+/-}$, $Zfp277^{-/-}$, $Apc^{Min/+}$, $Apc^{Min/+}Zfp277^{+/-}$, and $Apc^{Min/+}Zfp277^{-/-}$ mice. (**B**) Representative H&E staining of colon sections from mice with the indicated genotypes. (**C**) Representative H&E staining of small intestine sections from mice with the indicated genotypes. (**D**) H&E staining of Swiss rolls of small intestines (left) and colons (right) from mice with the indicated genotypes (proximal section at center). Size bars, 100 µM.

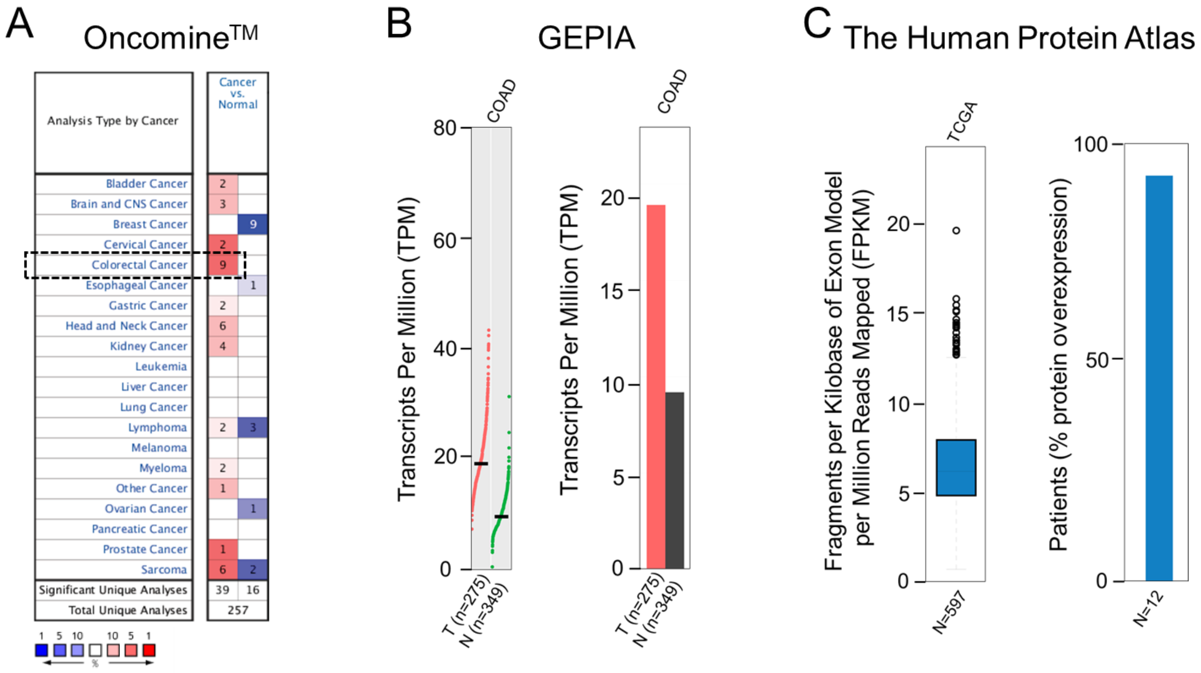

Supplemental Figure 4. ZNF277 mRNA and protein overexpression in colon cancer reported in public cancer databases. (**A**) Levels of *ZNF277* transcripts in colon cancer reported in the Oncomine^{TM} (**A**) and GEPIA (**B**) databases. (**C**) Levels of ZNF277 protein in colon cancer reported in the Human Protein Atlas database.

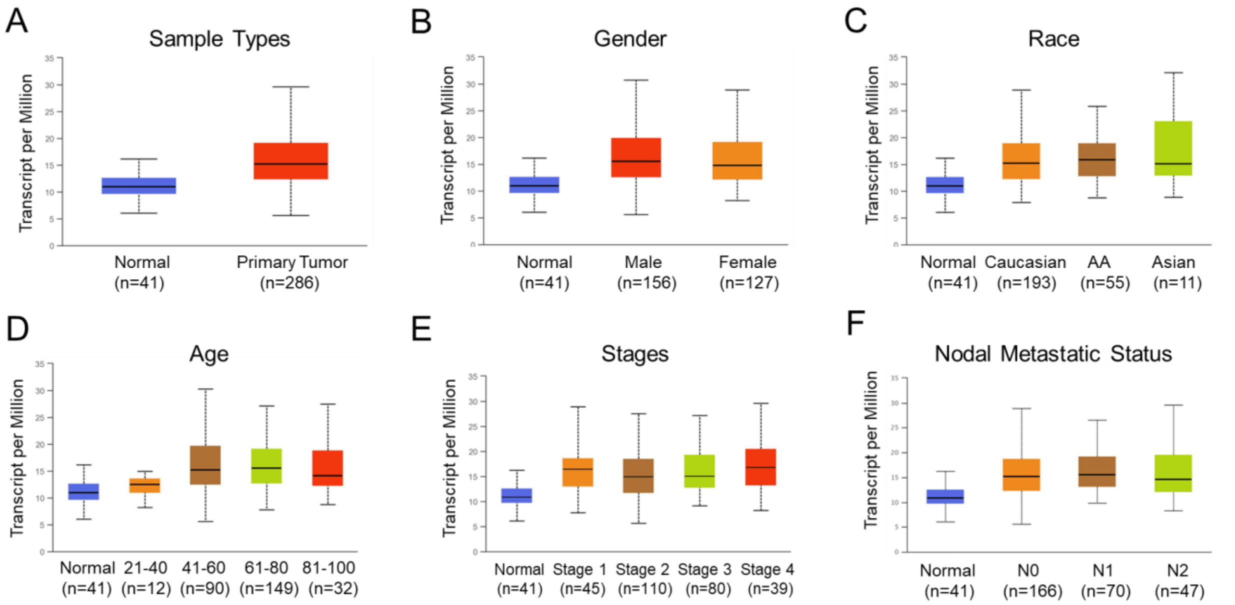

Supplemental Figure 5. Box-whisker plots showing *ZNF277* mRNA overexpression in all stages of colon cancer compared with normal colon reported in the UALCAN server analysis. *ZNF277* transcript levels are increased in primary tumors (**A**), and in tumors regardless of gender (**B**), race (**C**), Age (**D**), colon cancer stages (**E**) and lymph node metastasis status (**F**). All $P < 0.0001$. Student's *t*-tests.

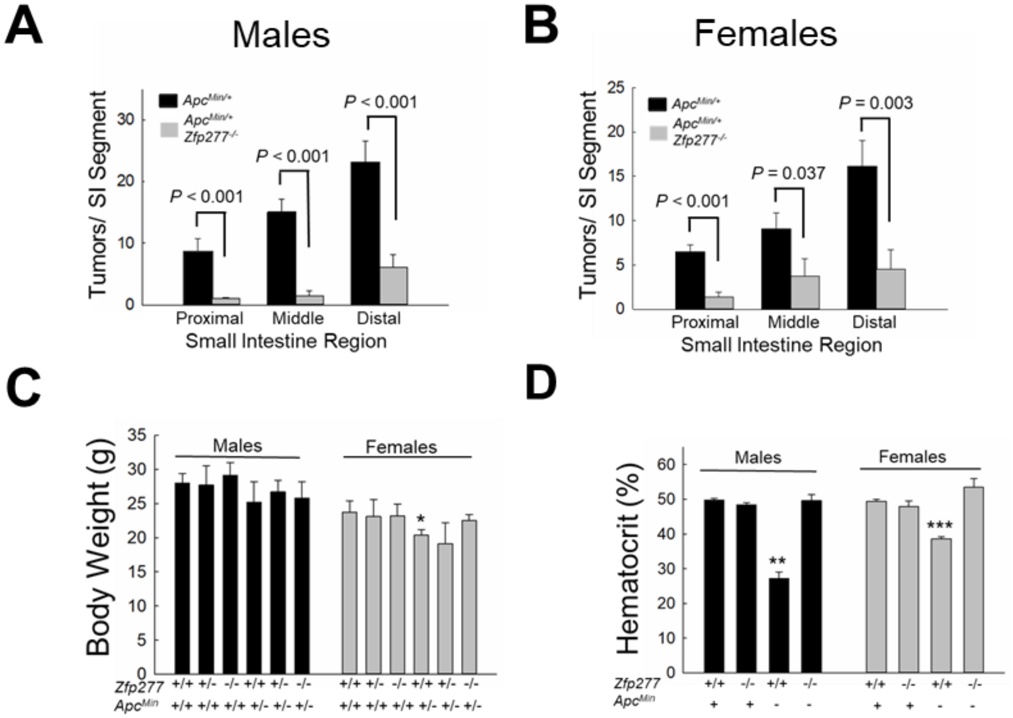

Supplemental Figure 6. Zfp277 deficiency attenuates small intestinal neoplasia, increases body weight and hematocrits in 15-week-old *$Apc^{Min/+}$* mice. (A and **B)** Numbers of small intestinal tumors in the proximal, middle and distal thirds of the small intestine of *$Apc^{Min/+}$* and *$Apc^{Min/+}Zfp277^{-/-}$* male **(A)** and female **(B)** mice. Male *$Apc^{Min/+}$* (n=12); Male *$Apc^{Min/+}Zfp277^{-/-}$* (n=10); Female *$Apc^{Min/+}$* (n=10); Female *$Apc^{Min/+}Zfp277^{-/-}$* (n=9). Values represent means ± SEM. Data were analyzed using the one- or two-tailed *t*-test or the Mann-Whitney *U* test. **(C)** Body weights of mice with the indicated genotypes. (**D**) Hematocrits of mice with the indicated genotype. Values represent means ± SD. One-tailed *t*-test. * P <0.05 vs. female *$Apc^{Min/+}$* mice; ** P < 0.001 vs. *$Zfp277^{-/-}Apc^{Min/+}$* mice; *** P = 0.002 vs. female *$Apc^{Min/+}$* mice. Body weights were measured in at least six 15-week-old mice of each genotype. Hematocrits were measured in three mice of each genotype.

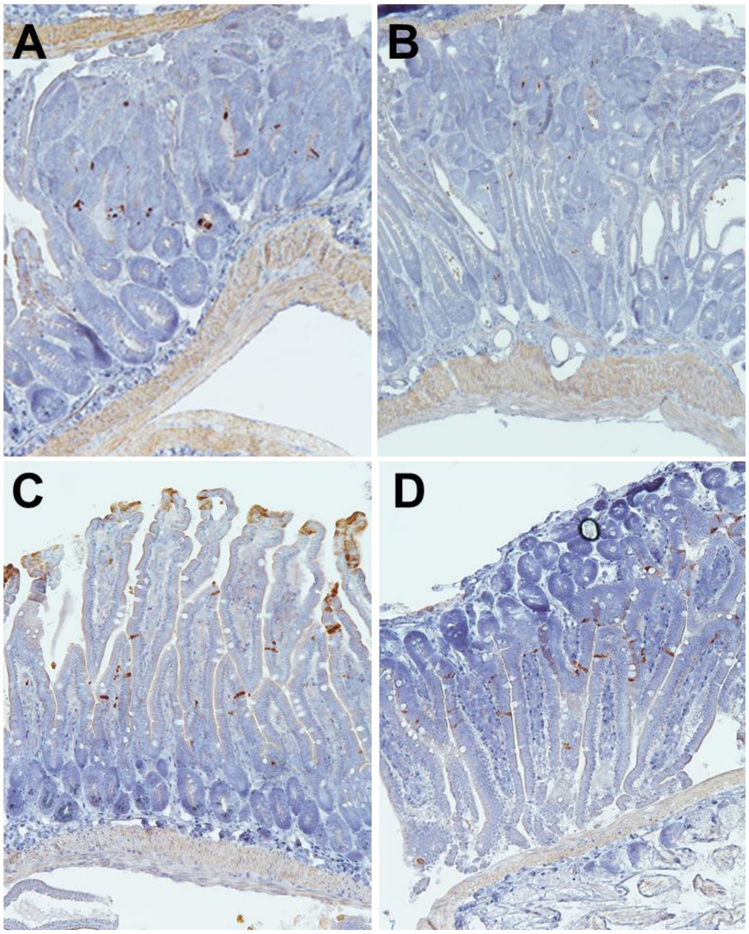

Supplemental Figure 7. *Zfp277* deficiency does not affect apoptosis. (A and **B)**. Images of cleaved caspase 3 IHC staining of small intestine adenomas from *Apc*$^{Min/+}$ **(A)** and *Apc*$^{Min/+}$*Zfp277*$^{-/-}$ **(B)** mice. **(C** and **D)**. Images of cleaved caspase 3 IHC staining of small intestines from *Apc*$^{Min/+}$ **(C)** and *Apc*$^{Min/+}$*Zfp277*$^{-/-}$ **(D)** mice.

Supplemental Table 1

Antibodies used in this study

Antibody	Catalog Number	Lot Number	WB (h)	WB (m)	HRP IHC	IF	ChIP
ZNF277	INV-PA555577				✓	✓	
ZNF277	PTG-25094-1-AP	21820	✓	✓			
BMI1	ABM-ab14389-25ug				✓	✓	
BMI1	CST #6964S	3	✓				
P16ink4a/cdkn2a	RND-NB200-106SS				✓		
p16INK4A	92803T	1	✓				
p21WAF1	RND-NBP2-29463-20ug	1026-		✓			
p21WAF1	CST #2947	11	✓				
p27KIP1	CST 3686S	5	✓				
p14ARF	CST #2407S	5	✓				
p15ink4b/cdkn2b	RND-MAB6798-SP						
p57KIP2	CST-2557T	2	✓				
Keratin (KRT) 20	CST-13063S	1			✓	✓	
β-Catenin	PMG-610153	7187864	✓	✓			✓
Ki67	INV-MA514520				✓	✓	
Ki67	PMG-550609				✓	✓	
Ki67	HPA000451-100UL				✓	✓	
p19ARF/CDKN2A Antibody	NB200-106SS						
Cleaved Caspase-3	CST-9664S				✓	✓	
Goat Anti-rabbit IgG , Alexa Fluor 555	INV-A21429				✓	✓	
Goat Anti-mouse IgG, Alexa Fluor 555	INV-A21422				✓	✓	
Goat Anti-rabbit IgG, Alexa Fluor488	INV-A11034				✓	✓	
Goat Anti-mouse IgG,Alexa Fluor488	INV-A11029				✓	✓	
Donkey Anti-rabbit IgG, Alexa Fluor 555	INV-A31572				✓	✓	
Donkey Anti-goat IgG, Alexa Fluor 488	INV-A11055				✓	✓	
GAPDH	sc-47724	D2117	✓	✓			
β-actin	PTG-60008-1-Ig	10004413	✓	✓			
Lamin B1	CST-13435		✓				
RNA Polymerase II	Thermo Scientific						✓
rabbit IgG	Thermo Scientific						✓
Mouse IgG	Thermo Scientific						✓

Supplemental Table 2

PCR Primer sequences

Genes	Forward primer sequence 5’ to 3’	Reverse primer sequence 5’ to 3’
ZNF277	ACAGCAGCAAGAACGAAATG	TGGCAATCCAATGTTGAAAG
p21^{WAF1}	GCGGTTGAATGAGAGGTTCC	AAGGAGAACACGGGATGAGG
Zfp277 Isoform 1	TACTGTGCCAGGTAATGGCTG	TTCGGAAATCTGCAACGAGC
Zfp277 Isoform 2	GTGCCAGGTAATGGCTGCTA	TGGCCATGTGGTTCAACAGT
HOXD13	CTTCGGCAACGGCTACTACA	CTTCTCCACGGGAAAGCCTC
ZNF277 ChIP 1	AGATCACTAAAGCATGTTCCCC	TGGTTGGGGAAAGTGGAGAT
ZNF277 ChIP 2	TGGCAAAGACTCCTCTGACA	AGTTTGACCTGAACCCACCT
ZNF277 ChIP 3	GGAGGAGGAAAAGGAAGGGA	AGGAACAGGATAGCTCTCGC
ZNF277 ChIP 4	TCACCCACGCCGTATTTCT	GGGACTGTGCATTGTTGTGA
GAPDH	CCCCATGGTGTCTGAGCG	CGACAGTCAGCCGCATCTT
Gapdh (m)	GGTGAAGGTCGGTGTGAAC	TGATGGCAACAATCTCCACT

Supplemental Table 3

Differentially-expressed Zfp277 target genes identified by RNA-Seq using murine colonic mucosa. Increased expression in red and decreased expression in blue.

Zfp277 Gene Pathways identified by DAVID	Gene names
Anterior/posterior pattern specification	Hoxd9, Hhex, Hoxc8, Wnt3, Msx1, Sfrp1, Ifitm1, Sfrp2, Hoxd13, Gli2, Hoxd10, Hoxd11
Pathway in cancer	Flt3, Gli1, Gli2, Hhip, Rasgrp3, Adcy7, Bdkrb2, Cxcl12, F2r, Csf1r, Csf3r, Ccne1, Fgf18, Fzd6, Fzd9, Lama2, Lef1, Mmp2, Mmp9, Nos2, Pdgfra, Rxrg, Spi1, Wnt3, Wnt4, Wnt8b
MAPK/ERK1/2	Ren1, Grin2B, Tlr13, Gdf10, Pik3ap1, Bmp5, Bmp8b, Bmp6, Fgf18, Ccl2, Ccl21a, Ptpn22, Ccl8, Ccl5, Cd74, Ccl11, Ccl12, Ccl22, Gm10591, C1qtnf3, Pdgfra, Gpnmb, Pla2g5, Csf1r, F2r
Canonical Wnt signaling	Fz9, Fzd6, Wnt4, Wnt3, Wnt8b, Daam2, Sfrp1, Sfrp2, Lef1, Ptpru, Snai2, Gli1, Cam2b
Cytokine-cytokine receptor interaction	Relt, Ccl11, Ccl12, Ccl2, Ccl22, Ccl3, Ccl4, Ccl5, Ccl8, Ccr10, Ccr2, Ccr5, Ccr6, Cxcl12, Cxcl14, Cxcr5, Cx3cr1, Csf1r, Csf2rb2, Csf3r, Il10ra, Il18rap, Il2ra, Il6ra, Il7r, Il9r, Lep, Lifr, Ltb, Gm10591, Gm1987, Tnfrsf13b

Supplemental Table 4

Differentially-expressed ZNF277 target genes identified by RNA-Seq in human HT29 cells. Increased expression in red and decreased expression in blue.

Top ZNF277 Gene Pathways and functional annotation clustering identified by DAVID	Gene names
Pathway in cancer	GNAI1, GNG4, KITLG, RASGPR2, WNT3A, WNT7A, WNT9A, WNT10B, WNT16, BMP4, F2R, COL4A3, COL4A4, COL4A5, COL4A6, CSF2RA, CCND1, CDKN1A ($p21^{WAF1}$), CYCS, EDNRA, FGF2, FGFR1, LAMA4, LAMC3, LPAR1, LPAR3, PTGS2, RET, STAT1, SMO
Signaling pathways regulating pluripotency of stem cells	LHX5, SMAD9, WNT3A, WNT7A, WNT9A, WNT10B, WNT16, BMP4, BMPR2, DUSP9, ESRRB, FGF2, FGFR1, LIFR, ONECUT1, PAX6
Proteoglycans in cancer	TIAM1, WNT3A, WNT7A, WNT9A, WNT10B, WNT16, CAV1, CAV2, CCND1, CDKN1A, EZR, FGF2, FGFR1, FLNC, ITGA5, PLAU, SMO
PI3K-AKT signaling pathway	GNG4, KITLG, CREB3L1, F2R, COL4A3, COL4A4, COL4A5, COL4A6, COL6A1, COL6A2, CCND1, CDKN1A, FGF2, FGFR1, ITGA5, LAMA4, LPAR1, LPAR3, OSMR, PPKAA2, PPP2R2C, SGK1
HOX (Homeobox)	LHX2, LHX5, LHX6, LMX1B, MEIS2, NKX1-2, NKX2-8, NKX6-1, TLX3, GBX2, HOXD8, IRX3, IRX4, MKX, MSX1, ONECUT1, ONECUT3, PAX6, PITX3, PRRX2, SHOX2, ZFHX4
